# Vulnerability in Children with Celiac Disease: Findings from a Scoping Review

**DOI:** 10.3390/children11060729

**Published:** 2024-06-14

**Authors:** Lúcia Macedo, Marta Catarino, Constança Festas, Paulo Alves

**Affiliations:** 1 Âncora Community Care Unit, Gaia and Espinho Local Health Unit, 4430-037 Gaia, Portugal; 2 Institute of Health Sciences (ICS), Universidade Católica Portuguesa, 4169-005 Porto, Portugal; cfestas@ucp.pt (C.F.); pjalves@ucp.pt (P.A.); 3 Center for Interdisciplinary Research in Health (CIIS), Institute of Health Sciences (ICS), Universidade Católica Portuguesa, 4169-005 Porto, Portugal; marta.catarino@ipbeja.pt; 4 Health Department, Polytechnic Institute of Beja, 7800-111 Beja, Portugal; 5 Institute of Health Sciences (ICS), Universidade Católica Portuguesa, 1649-023 Lisboa, Portugal

**Keywords:** children, celiac disease, vulnerability, nursing, scoping review

## Abstract

(1) Background: The scientific literature highlights that children diagnosed with celiac disease (CD) are at a heightened risk of experiencing physical, psychological, and social challenges, impacting their overall healthy childhood development. However, there remains a lack of a clear understanding regarding the factors that contribute to this vulnerability. The purpose of this study is to analyze and map the evidence on the sociopsychosomatic vulnerability of these children and identify gaps in this topic. (2) Methods: Following Joanna Briggs Institute’s guidelines for scoping reviews, we executed a detailed search of key electronic databases and explored the grey literature to capture a broad spectrum of studies. Our focus was on identifying research that looked into the multiple dimensions of vulnerability—physical, psychological, and social—in children with CD. We included a diverse range of study designs as well as systematic reviews, ensuring a comprehensive analysis. The selection process was stringent, utilizing clearly defined inclusion and exclusion criteria. (3) Results: We identified 61 studies that met our inclusion criteria. The review highlighted significant adverse health outcomes in children with CD and elucidated various individual and environmental determinants that influenced these vulnerabilities. It also underscored the lack of assessment tools to evaluate the risk of health problems in this population. (4) Conclusions: The findings underscore a critical need for further research to deepen our understanding of the vulnerabilities associated with CD in children. Developing targeted assessment tools will be crucial in stratifying health risks and enhancing care strategies for this vulnerable population.

## 1. Introduction

The experience of childhood with the management of a chronic health condition involves a nuanced journey that requires continual adaptation and adjustment. A child diagnosed with celiac disease (CD) might face an elevated risk of various disorders (physical, psychological, and social), diminished quality of life, and heightened stress compared to their healthy counterparts, stemming from the genetic predisposition associated with the condition [1,2,3,4,5,6].

CD is a multifaceted, systemic, immune-mediated disorder that can be diagnosed at any stage of life. The condition is influenced by various factors, including the individual’s HLA immunogenetic background, notably the presence of DQ2 and DQ8 heterodimers, particularly HLA-DQB1*02, as well as environmental triggers such as gluten. It’s noteworthy that while both factors are essential, they alone are insufficient to precipitate the development of CD [2,4,5]. The etiology of CD is not fully understood. Genetic susceptibility is a prerequisite, with the possible participation of other environmental cofactors [7].

Recent epidemiological studies have suggested that the prevalence of CD is at least 1:100, with a range between 1–2% across different countries globally, although the true prevalence is difficult to ascertain due to significant underdiagnosis. The average duration of diagnosis is 10 years [2,8,9,10]. Advancements in understanding the pathogenesis, enhancements in diagnostic methodologies, and heightened awareness over time have revolutionized our comprehension of CD. It has evolved from being perceived as a rare enteropathy to a prevalent multisystem disorder affecting individuals across all age groups, with a spectrum of clinical presentations. Clinical manifestations can range from overt intestinal symptoms to asymptomatic disease. Now, it’s considered to be a “clinical chameleon” [11]. Only a minority of children now present with the classical clinical picture of profound diarrhea and malnutrition. [12,13].

Although there is ongoing research to develop medications that could help treat CD, however, so far, no medication has been approved as a substitute for the GFD. The only (but also effective) treatment for CD is a strict, lifelong gluten-free diet (GFD), a regimen accompanied by perceived treatment complexities and psychosocial implications [3,8,12,14]. The presence of mental-health comorbidities and diminished quality of life have been correlated with decreased adherence to a GFD, subsequently heightening the susceptibility to health-related complications [1,2,3,6,15].

We hypothesize that individuals have a vulnerability threshold resulting from the interplay of personal and environmental factors. When this threshold is exceeded, health problems emerge. Thus, understanding vulnerability before health issues arise is of significant interest [16]. Although some current reviews focused on the genetic role (and, therefore, seeing an evolution of diagnostic techniques and treatment) in the development of disease and associated health problems [4,5,12,17], quality of life, experiences, and difficulties of children with CD (and families) have received more attention from researchers. However, the ratio of articles devoted to CD diagnosis versus those dealing with the care of patients is >10:1 [18].

To date, little attention has been focused on variables (individual/environmental) that determine a person’s vulnerability, influencing their health status in various dimensions (physical and psycho-social). The scoping review was our method of choice because of the exploratory nature of our research question. This study will review scientific evidence on vulnerability in children with CD across care settings, identifying gaps and informing future research needs [19].

## 2. Materials and Methods

This scoping review was designed to comprehensively map the existing literature on the vulnerabilities associated with CD in children, encompassing a broad conceptual range in line with our study objectives.

This scoping review was guided by the Joanna Briggs Institute’s (JBI) methodology for scoping reviews. The methodology involved several structured steps: formulating precise research questions, developing a robust article search strategy based on relevant keywords, and carefully selecting studies through a systematic screening process. Each article was then analyzed to extract and synthesize data relevant to the vulnerabilities in children with CD. Our review adhered strictly to the Preferred Reporting Items for Systematic Reviews and Meta-Analyses guidelines for Scoping Reviews (PRISMA-ScR) [20,21], which guided the selection and identification process for the literature on various aspects of vulnerability.

A preliminary search across MEDLINE (PubMed), the J.B.I. Evidence Synthesis, the Cochrane Database of Systematic Reviews, PROSPERO, and Open Science Framework (OSF) did not reveal any systematic literature reviews or ongoing scoping reviews on this topic [19,22,23].

Additionally, this review was registered on the OSF (https://doi.org/10.17605/OSF.IO/PXS34, accessed on 22 March 2024), ensuring transparency and accessibility of our research process and findings.

### 2.1. Study Design

The study design followed a systematic and rigorous approach to ensure the comprehensiveness and quality of the scoping review. This methodology makes it possible to map the extent, variety, and nature of the evidence available on the topic, providing a solid basis for future research.

### 2.2. Review Question

The review question emerged through the mnemonic P (population—child or adolescent with CD), C (concept—vulnerability), and C (context—healthcare): “*What evidence has been published regarding the vulnerability of children or adolescents with CD in different health care settings?*”

Furthermore, this review intended to answer the following sub-questions:

1. What is the published evidence on the different dimensions of vulnerability in children with CD?
2. What other concepts are related to the concept of vulnerability in children with CD?
3. Is there evidence on the use and/or validation and/or development of assessment tools on vulnerability?
4. Is there published evidence on determinants that, positively or negatively, influence vulnerability in children with CD?

### 2.3. Elegibility Criteria

#### 2.3.1. Participants

The review specifically targeted studies that included children with CD spanning various developmental stages—from preschool through adolescence. Our age criteria for participants were aligned with the definitions provided by the Convention on the Rights of the Child, which categorizes individuals under 19 years of age as children, and the World Health Organization’s classification, which defines children as those aged 10 to 19 years [24,25]. We included studies involving children from the early educational stage (preschool) up to the end of secondary education (third cycle), effectively covering ages 3 to 18 years. The review did not impose any restrictions regarding the demographic characteristics of the study populations, such as gender or ethnicity, to ensure a comprehensive analysis of available data without bias.

#### 2.3.2. Concept

In this study, citations that addressed their vulnerability were included.

#### 2.3.3. Context

Studies that addressed vulnerability in children and adolescents with CD, involving their caregivers, the family environment, and the different contexts of health care (mainly community care and school health) were included.

#### 2.3.4. Types of Studies

The review comprehensively included quantitative studies encompassing descriptive, observational, analytical, experimental, and experimental analytical methodologies. Qualitative studies were also incorporated, covering diverse designs such as documentary research, case studies, ethnography, and phenomenology. Additionally, studies employing mixed-methods approaches and relevant grey literature were considered to ensure a broad representation of the data available on the subject.

To maintain the scientific rigor of our analysis, certain types of publications were excluded from the review. These exclusions encompassed editorials, letters to the editor, protocol reviews, and study abstracts, as these documents typically do not provide comprehensive data or detailed research methodologies essential for a through scoping review.

### 2.4. Research Strategy

The research strategy was designed to identify both published and unpublished primary studies and reviews. Two reviewers developed this strategy, which was then rigorously peer-reviewed by a third expert based on the Peer Review of Electronic Search Strategies (PRESS) guidelines established by McGowan in 2016 [19].

We employed the three-step search strategy recommended by the Joanna Briggs Institute (JBI) [22]. Initially, a preliminary limited search was conducted on MEDLINE via PubMed to explore key terms within the titles, abstracts, and index terms related to the studies. This step helped refine the search terms to ensure comprehensive coverage of the relevant literature. Following this, a full search strategy was formulated, incorporating indexed terms, synonymous terms, truncations, and Boolean operators (AND, OR). This search was meticulously carried out across the fields of subject, title, abstract, and keywords on 8 May 2023, to ensure all pertinent studies were included.

Thus, the following research equation was obtained: (“vulnerab*” OR “risk” OR “susceptib* AND (“celiac disease” OR “coeliac disease” OR “celiac sprue” OR “gluten-sensitive enteropathy”) AND (Child* OR adolescen* OR infan* OR teen* OR youth OR scholar OR pediatric OR paediatric).

It´s emphasized that the search strategy was adapted to the specificities of each information source. Finally, the reference lists of the articles included in the review were selected for supplementary articles.

Studies published in English, Spanish, Castilian, French, and Portuguese were included, without time limitations, in the following databases: Scopus by Elsevier, 1975–2023; Web Of Science by Clarivate, 1988–2023; MEDLINE by EBSCO Host, MEDLINE by PubMed, 1991–2023, CINAHL Complete by EBSCO Host, 1992–2023, Cochrane Central Register of Controlled Trials, by EBSCO 1996–2023, SciELO—Scientific Electronic Library Online (1997–2023) and Psychology & Behavioral Sciences Collection, via EBSCO Host (2002–2023). The search for unpublished studies in the gray literature included the Scientific Open Access Scientific Repositories of Portugal (RCAAP) database.

The investigation strategy is described in detail in Appendix A (Table A1).

### 2.5. Selection of Studies

After the search, all identified citations were exported to the EndNote Web software (X9) (Clarivate Analytics, Philadelphia, PA, USA) [26]. Duplicates were eliminated during this process. The study selection occurred in two phases, facilitated by the Rayyan QCR platform [27]. Initially, titles and abstracts were reviewed, and studies not meeting the eligibility criteria were excluded. This screening was conducted independently by two investigators. In the second phase, the full texts of potentially relevant citations were meticulously evaluated by the same two investigators independently.

### 2.6. Data Extraction

The data were independently extracted from the works included in the scoping review by two reviewers. A structured instrument, defined by the JBI [22], (Table A2 in Appendix B) was used, where the following information was transferred: author/s; year; country; title; type of study; population; dimensions/issues/characteristics of vulnerability in children with CD; concepts related to the concept of vulnerability in children with CD; tools to assess vulnerability; and situations, circumstances and conditions/factors/determinants that positively or negatively influence vulnerability in children with CD.

### 2.7. Data Analysis and Presentation

The data collected was summarized in schematic and tabular formats, in accordance with the JBI recommendations [21], through consensus reached by two researchers. The tabular presentation summarizes the key information and is complemented by a narrative summary, discussing the results and elucidating their relevance to the research objectives and questions.

## 3. Results

### 3.1. Characteristics of Included Studies

A total of 8658 records were obtained in the search performed on 8 May 2023: 1575 from MEDLINE Complete by EBSCO Host, 365 from CINAHL Complete by EBSCO Host, 26 from Psychology and Behavioral Sciences Collections by EBSCO, 1778 from MEDLINE by PubMed, 2880 from Scopus by Elsevier, 1953 from Web of Science by Clarivate, 68 from Cochrane Central Register of Controlled Trials, 10 from SciELO, and 3 from Scientific Open Access Scientific Repositories of Portugal (RCAAP). A total of 4778 studies were excluded due to duplication. Two independent researchers analyzed the remaining 3880 articles. The title and abstract were reviewed, resulting in the exclusion of 3818 studies, allowing for the evaluation of 62 articles for eligibility.

**Figure 1:**
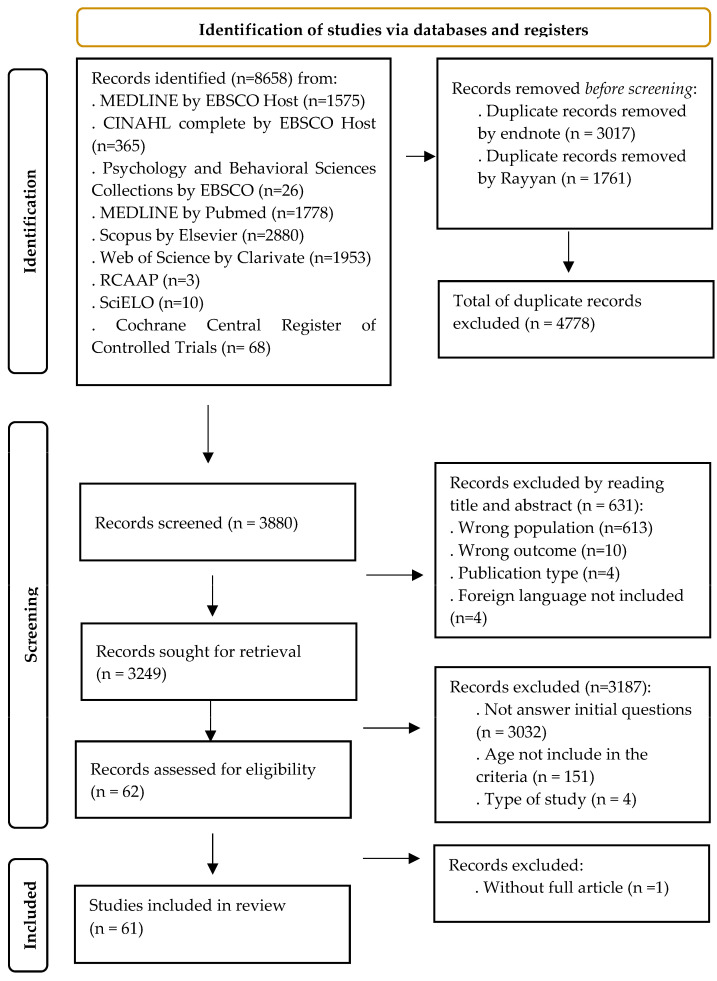
PRISMA of study selection and inclusion process [21].

The final selection of studies was based on confirming the selection and exclusion criteria, following individual reviews and investigator meetings. Out of the 3187 articles, 3032 were excluded as they did not address the initial research question. Additionally, 151 studies did not meet the specified age criteria, 613 studies focused on inappropriate populations, and 10 studies did not align with the review objectives. Furthermore, 4 studies were excluded due to being protocol reviews, and 4 were excluded for being published in languages not included in the criteria. Consequently, a total of 61 studies were included in the analysis. The data collection and selection processes are depicted in Figure 1.

Studies were published between 1998 and 2023, although predominantly in recent years. They were published in 24 different countries, mostly in Europe (54.1%; *n* = 33). There were 15 studies from America, followed by Asia with 12 studies, and Australia with 1 study.

The implications of CD for children and families have been studied by various disciplines: medicine (*n* = 46); nursing (*n* = 3); nutrition (*n* = 3); psychology (*n* = 3); public health (*n* = 3); dentistry (*n* = 1); pharmaceutical and health sciences (*n* = 1); and anthropology (*n* = 1) (Table A3—Characteristics of the articles included in this scoping review in Appendix C).

Of the 61 studies included in this scoping review, 43 were primary studies (70.49%). Most studies, 62.29% (*n* = 38), were quantitative studies (cross-sectional, retrospective, and prospective, cross-sectional studies), 21 were qualitative studies (phenomenological, qualitative interviews, and systematic and narrative studies) and 2 were mixed studies.

Most of the population included in the studies were only children/adolescents with CD (*n* = 48), children with CD and their parents or caregivers (*n* = 4), only parents or caregivers of children with CD (*n* = 4), adults with CD diagnosed in childhood (*n* = 1), children with T1DM and CD (*n* = 2), only parents or caregivers of children with T1DM and CD (*n* = 1), and web pages related to GF parenting and GF families (*n* = 1).

The characteristics of the included studies are summarized in Appendix C (Table A3).

### 3.2. Review Results

With regard to question 1 (What is the published evidence on the different dimensions of vulnerability in children with CD?), although all the studies included looked at the impact and the repercussions of CD on children’s health, they mostly used a concept related to risk or fragility as there are similarities in the application of the concept with regard to vulnerability.

Thus, we included studies with results that matched the definition of vulnerability proposed by Rogers.

Of the 61 studies included, 27 authors reported adverse implications/outcomes for the child’s physical health, 18 mentioned consequences for mental health, 7 for social life, and 2 for society in general. Several adverse health outcomes have been identified, as shown in Table 1.

Appendix C (Table A3) reported the data presentation template for Question 1.

**Scheme 1:**
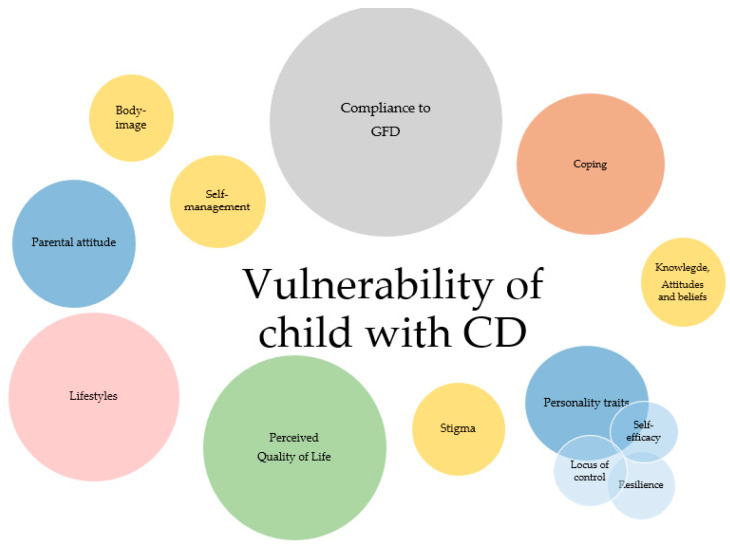
Representation of concepts related to vulnerability in children with CD.

Regarding Question 2 (What other concepts are related to the concept of vulnerability in children with CD?), the concepts most referred to in the studies were compliance with a GFD (*n* = 39) and QoL (*n* = 18) followed by lifestyles (*n* = 16). Others were mentioned less often: coping (*n* = 8), parental attitude/parental stress (*n* = 5), stigma (*n* = 3), self-management (*n* = 3), personality traits: LoC, self-efficacy, resilience (*n* = 5), knowledge, attitudes and beliefs (*n*= 3), and body-image (*n* = 3). Scheme 1 and Table A3 described the data presentation model for Question 2. Appendix C summarizes the studies addressing concepts related to vulnerability (Table A5).

Regarding Question 3 (Is there evidence on the use and/or validation and/or development of assessment tools on vulnerability?), several studies have addressed the adverse outcomes (or variables) considered most significant for the risk of developing complications (or adverse health outcomes), demonstrating their health vulnerability. Several studies (*n* = 15) used different assessment instruments according to their study objectives, but no tool has been found that assesses (or stratifies) vulnerability in children (Appendix C Table A3).

Regarding Question 4 (Is there published evidence on determinants that, positively or negatively, influence vulnerability in children with CD?), various studies have shown the contribution of different individual (biological, sociodemographic, personality traits, factors related to the disease itself, and acquired) and environmental (family, community, and society) variables (Table 2).

Appendix C (Table A6) reported the data presentation template for Question 4.

## 4. Discussion

Although acknowledging the evolution of the concept of vulnerability over the last three decades, the authors adopted Rogers ‘definition, which conceptualizes vulnerability as resulting from the dynamic interaction between individuals’ personal resources and the environmental support available to satisfy their health needs [84]. This pragmatic and comprehensible model is appropriate for implementation in the context and characteristics of the population being investigated. This scoping review appears to be the inaugural study that systematically compiled and discussed the vulnerability of children with CD. Our review aimed to thoroughly map the existing literature to discern determinants that influence vulnerability in these children, either positively or negatively. We also sought to identify associated concepts and tools for assessing vulnerability, thereby addressing significant gaps in the current understanding.

The findings from this review were based on knowledge from multiple disciplines including nursing, medicine, nutrition, public health, psychology, anthropology, dentistry, pharmaceutical sciences, and health sciences. This interdisciplinary approach has provided a robust framework for supporting and understanding the vulnerabilities associated with CD in children.

Particularly within the discipline of nursing, our findings underscored the critical role of health education in managing CD, especially within elementary educational settings. School-age children with CD encounter numerous challenges in adhering to a strict gluten-free diet, a fundamental component for preventing complications and ensuring quality of life into adulthood.

There is a general consensus in the literature that children with CD are at a heightened risk of developing multiple health complications compared to their non-CD peers. Our review highlighted the broad implications for physical, psychological, and social health as well as the potential long-term societal repercussions (see Table 1 for detailed implications).

### 4.1. Vulnerability to Physical Health of Children with CD

The diagnosis of CD marks the beginning of a lifelong gluten-free journey. A GFD is the only treatment available, which is indispensable for controlling the disease, healing the mucosa, and preventing complications [28,85]. Consensus suggests that sustained consumption of gluten, even in minimal quantities, can induce chronic inflammation and malabsorption, with gluten-related manifestations affecting various organs, particularly growth. In addition, iron deficiency anemia is among the most prevalent laboratory abnormalities observed in CD patients [86,87].

Growth failure, bone health impairment (and reports of skeletal problems), and delayed onset and disordered progression of puberty associated with CD have been documented for a long time [4,28,88,89,90,91]. However, the contemporary literature indicates that individuals diagnosed in childhood increase their bone density more rapidly [92], and normal bone mineral density is achieved with long-term adherence to a GFD [36,93,94]. Furthermore, research suggests that children diagnosed and treated before puberty can reach normal peak bone mass, potentially avoiding osteoporosis later in life [4,28,86,95,96].

Recent studies suggest the development of increased weight in CD patients may be also influenced by the global trend toward overweight and obesity in pediatric populations [97]. Moreover, numerous reports have explored the association between a GFD and obesity in youths with CD, with conflicting results [45,98,99,100,101,102,103,104,105].

Diet quality plays a crucial role in children’s optimal growth and development and in their long-term health. Although adherence to a gluten-free diet is crucial to the success of treatment and the prevention of other complications, recent studies indicated that patients who adhere to a GFD may be susceptible to nutritional deficiencies. This vulnerability results from the adoption of less nutritious eating habits associated with the consumption of highly processed gluten-free products, which are often deficient in fiber, iron, vitamin D, and calcium, and have a high glycemic index [33,51,52,106,107]. Studies reported that 84% of children and adolescents eat GF products (with greater carbohydrate and lipid content than their gluten-containing equivalents to improve food palatability and consistency) between two and three times a day [42,106].

Maintaining a GFD may promote a less healthy diet by substituting gluten-containing foods with ultra-processed foods [108]. Thus, there is evidence to suggest that a GFD may, therefore, have a negative impact on cardiometabolic risk factors (such as obesity, serum lipid levels, insulin resistance, metabolic syndrome, and atherosclerosis) [46]. A systematic review and meta-analysis concluded CD was associated with a modestly increased risk of cardiovascular disease, but the evidence base is limited [109].

The study highlights the importance of dietary counseling for celiac patients. This counseling serves as a fundamental tool to educate patients on increasing consumption of naturally GF products, minimizing processed foods, incorporating cereals such as oats, rice, minor grains, and pseudo-cereals, and adhering to the principles of the Mediterranean diet [47]. It is a well-known clinical spectrum of CD, remarkably varied, often associated with other autoimmune disorders, affecting many extra-intestinal organs and systems [50]. It is agreed that T1DM and hypothyroidism were the most commonly associated autoimmune conditions in children with CD [79,110,111]. Each of these diseases affects 4–5% of the population with CD [112,113,114,115,116]. The association between CD and autoimmune thyroiditis has been consistently documented in pediatric populations [4,117,118,119,120,121,122,123]. Another study found that the elevated risk of epilepsy is attributable to an increase in epilepsy diagnoses coinciding with the diagnosis of CD. After stratification by sex, a remarkably high risk of epilepsy was observed among females with CD. Sensitivity analyses corroborated the positive association between CD and epilepsy [35].

Recent studies indicated that the overall prevalence of extra-intestinal manifestations is compared between pediatric and adult populations. However, the prevalence of specific manifestations and the rate of improvement varies between age groups. The pathogenesis of extra-intestinal manifestations remains incompletely understood. Two main mechanisms have been proposed: the first is linked to malabsorption resulting from mucosal damage and the second is associated with a sustained autoimmune response [124].

### 4.2. Vulnerability to Mental Health of Children with CD

Although the etiopathogenesis of psychiatric signs/diagnoses in CD remains unclear, tryptophan deficiency resulting from malabsorption in patients with poor adherence to the diet may induce a hyposerotonergic state in the central nervous system. Additionally, psychosocial stress associated with CD and adherence to a GFD is considered another significant factor. There is a consensus among various researchers [59,64,66,72].

In contrast to many chronic medical conditions managed primarily through pharmaceutical interventions, a diagnosis of CD necessitates a significant lifestyle adjustment for the patient. This adjustment includes constant attention to ingredient lists, restaurant menus, and social situations due to the pervasive presence of gluten in socializing and everyday life. Research indicates that the stress associated with this lifestyle change may contribute to a higher incidence of psychiatric disorders in both the short and long term [68,125].

Eating is a vehicle for satisfying social and relational needs between individuals, incorporating a social and even political dimension. Consequently, a diet that restricts opportunities for dining out, traveling, and other social activities becomes a significant impediment to socializing, limiting access to social opportunities [126,127]. Also, individuals who avoid certain foods due to allergies or intolerances, even when medically necessary, run the risk of being seen as picky eaters, self-centered, politically marginal, demanding, or old-fashioned [128].

Studies have revealed that children with CD, limited by dietary restrictions, often experience feelings of loneliness as a result of their limited ability to relate to other people at mealtimes. These studies indicate that children may feel uncomfortable being singled out as "different" because of the attention given to their eating condition. In addition, research suggests that CD is associated with higher levels of psychological distress, including depression, anxiety, and social phobia. The resulting discomfort in school and social life can lead to vulnerability, isolation, and stigmatization [4,59,129]. These children are particularly affected by social stigma; they have to renounce eating what their peers consume during school breaks or at birthday parties and often take their "special" tray to social gatherings [127].

The cultural, emotional, and psychological significance of food and eating makes CD a pathology with a profound psychological impact. Food is interlinked with rituals, traditions, festivities, and religions, becoming a channel for conscious and unconscious meanings capable of influencing an individual’s entire lifestyle [73]. It is expected that these situations can lead to feelings of low self-esteem and constitute a risk factor for psychopathology [4,59].

As with other chronic diseases, studies suggest that psychiatric morbidity is likely to contribute to lower adherence [74]. Several cohort studies have indicated that untreated CD is associated with psychiatric morbidity. The greatest excess risks for depression and anxiety were observed immediately before and 4–8 years after the diagnosis of CD. Therefore, caregivers should consider this and involve the whole family when supporting a patient with CD [130].

Children with CD have an increased risk for various psychiatric disorders, potentially attributed to the biological and/or psychological effects of CD [131]. In addition, the risk of suicide is higher among CD patients compared to controls from the general population [132]. Several authors have acknowledged the potential relationship between CD and the risk of developing eating disorders (EDs). This connection may be attributed to the need for patients with CD to be vigilant about food availability and cross-contamination, as they must avoid prominent gastrointestinal symptoms associated with the pathology. Such vigilance may contribute to a fear of eating and could potentially lead to disordered eating attitudes and behaviors [64,65,66,76]. Disordered eating behaviors (DEBs) are prevalent during adolescence and young adulthood and represent a risk of progressing into full-blown eating disorders. Studies have identified female gender, older age, and being overweight as risk factors for DEBs in individuals with CD [64,65].

Evidence indicates that chronic illness requires a restructuring of family dynamics to accommodate the internal and external changes resulting from the illness and the associated care demands [73]. Consequently, a child’s chronic illness may also influence parents’ psychological adjustment. [133]. A study conducted in Italy revealed that parents of children with CD experienced higher levels of parental stress compared to parents of healthy children [73]. These studies also highlighted various challenges faced by parents, including managing symptoms of the disease, navigating the diagnostic process, regulating the diet, addressing difficulties encountered at school and in social environments related to dietary restrictions, ensuring access to appropriate food for the diet, dealing with economic concerns, and anticipating future challenges [61,69].

Studies suggested parental psychosocial problems may have an effect on the physical health of a child with a chronic illness in addition to influencing the psychosocial functioning of the child. Having a child with CD might have negative effects on mothers and their attitudes toward their children, including higher levels of depression and anxiety, excessive motherhood, and strict discipline [72]. Also, previous studies suggested that parents of children with chronic illnesses are overprotective, have less close and positive relations with their children, and that the severity of the chronic illness leads to more frequent protective and controlling behaviors in the parents [134,135,136]. More efficient parenting helps to decrease the behavioral problems of children [72].

Managing both CD and DM1 in a child can be particularly challenging due to the specific management requirements that can impact the health outcomes of each condition. This situation can be emotionally, psychosocially, and financially demanding for parents [55,137,138].

For all these reasons, in view of the results found, it becomes imperative to value the psychological/emotional aspects and the social impact of monitoring these children in order to optimize therapeutic intervention [139] as well as assess the QoL perceived by the child/family.

### 4.3. Vulnerability in the Social Life of Children with CD/Family and Its Impact on Society

Numerous studies have emphasized that adopting a GFD imposes substantial lifestyle changes for families. CD demands a profound alteration in eating habits for both patients and their families, requiring meticulous management in food procurement, preparation, and storage. This adjustment can have a significant impact on various aspects of life, including career and family dynamics, social anxieties, and a feeling of being different [3,71,140,141].

Furthermore, research has shown that a child’s diagnosis of CD affects multiple family members, including mothers, fathers, and siblings, who in turn influence the child with CD. Mothers often bear the brunt of caring for their children’s dietary needs, both qualitatively and quantitatively [54,142]. The maintenance of a child’s GFD places considerable physical, emotional, relational, and mental burdens on mothers [54]. In some instances, the literature has described mothers as being unfairly labeled as overbearing, a stereotype stemming from societal criticism of intensive, risk-averse mothering. Nevertheless, mothers are still expected to adhere to intensive parenting ideologies [54]. Fathers typically report experiencing less overall burden than mothers; although, they are also affected by CD, such as through limited restaurant options. Siblings, whether affected by CD or not, also face constraints in food choices [142].

Studies showed mothers predominantly bear the responsibility for maintaining a safe environment for their child within the home and assume the majority of the caregiving duties [142].

A strict, lifelong adherence to a GFD remains the only treatment for children with CD. Compliance with a strict GFD was associated with decreased mortality rates from the disease. However, mortality risks were heightened due to external causes and malignant diseases, particularly gastrointestinal malignancies, in CD patients, particularly those who did not adhere to a GFD [74,141,143,144].

Adherence to a GFD in CD is crucial for improving health outcomes, resolving symptoms, and ensuring a normal life expectancy without disease complications. Poor adherence increases the risk of persistent short- and long-term complications [4,36,49,55,83].

By identifying the variables that affect adherence to a GFD, we can understand the variables that affect vulnerability in children with CD, since vulnerability is the adverse result of non-adherence.

Knowledge of these determinants is essential to the development of interventions aimed at improving it and decreasing their vulnerability.

### 4.4. Individual Determinants of Vulnerability in Children with CD

Of the individual determinants, biological variables stood out, namely the sharing of a genetic background [51,52], which potentiates the appearance of other diseases: T1DM/ATD [49,52,63,75]. The shared genetic background of T1DM and CD, primarily attributed to the presence of HLA class II genes such as DQ2 and DQ8, has been extensively documented. These genes are present in 95% of patients with T1DM and nearly 99% of celiac patients, significantly elevating the risk for both diseases compared to the unaffected population [4,63,145,146,147,148,149,150].

For a long time, the association of these two pathologies was studied, assuming that T1DM would appear before CD—which, in fact, happens in approximately 2/3 of cases [146]. Some studies have subsequently emerged suggesting that the order in which these diseases appear could be reversed [149]. Among other possible justifications was the fact that CD is often oligosymptomatic or silent and could, therefore, have existed before DM1, but had not yet been diagnosed. Some studies on the CD–T1DM association show a prevalence of 3.6–3.9% of T1DM in patients with CD [51,52,63,116,151].

Both T1D and CD present long- and short-term health complications that need to be prevented or managed, making this subgroup of children especially vulnerable [55,152].

The most problematic aspect for a child with T1DM and CD is that most GF foods have a high glycemic index, while low glycemic index foods are recommended for T1DM, demonstrating the increased vulnerability and impact on quality of life resulting from these two comorbidities. Specialized follow-up and dietary counseling are essential in the management of patients affected by both T1DM and CD [51,52,55,152].

Studies have shown a higher prevalence of CD among females [34], with females exhibiting a higher prevalence compared to males [153]. Additionally, research indicates that emotional functionality and physical health are more affected in girls [59].

Childhood and adolescence are considered vulnerable periods for individuals. Adolescence, in particular, is seen as a critical age for dietary compliance. This age group has been the primary focus of numerous extensive European studies concerning the psychosocial impact of CD [81,154,155,156,157].

The provision and management of care during adolescence represents a delicate phase, influenced by the greater vulnerability of adolescents when faced with the challenges of their age and illness, often resulting in decreased adherence to the GFD [18,33,70,76,77,81,127]. Adherence to the GFD is a challenge at all ages, but is particularly difficult for adolescents due to social, cultural, economic, and practical pressures, as consistently reported in several studies [18,76]. Notably, age showed a significant negative correlation with diet adherence, even after adjusting for other confounding factors.

While young children with CD usually adhere to a GFD due to parental influence, the situation becomes more complex in adolescents [158]. Parents are often less involved in food decisions and supervision of their children, especially in social contexts involving meals or snacks [18,77,81,159]. This trend was supported by numerous studies that identify factors contributing to non-compliance, such as increasing age, intensified social interactions, peer pressure, increased outdoor activities, and the desire to experiment [83,158,160,161,162].

Many adolescents maintain a GFD at home but intentionally consume gluten-containing foods in social settings, whether at home or at parties [18,127,163]. Reasons for these transgressions often included issues related to GF product availability, cost, palatability, social events with friends, voluntary choice, and a desire to conceal the problem for social acceptance [127,163,164]. Consequently, teenagers with CD may face risks to social integration, self-esteem, and academic achievements, which may exceed clinical complaints [18]. While compliance with a GFD tended to be high when initiated in childhood, the percentage of adolescents with CD who strictly adhered to a GFD varies widely, ranging from 40% to 81% [76,107,164]. Higher adherence rates were typically observed among patients diagnosed in childhood and those presenting with severe gastrointestinal symptoms [165,166]. However, studies have noted a decrease in dietary compliance among children above 9 years of age, with compliance rates dropping from 75.92% in children aged >2–5 years to 41.37% in those above 9 years [158]. Similarly, adherence rates declined from 93% at 12 years of age to 76% in the age group 15–17 years [160].

Despite comparable adherence rates between girls and boys, women with CD may face greater challenges, reporting a more significant impact of the disease on their social interactions and participation in significant life events [80,126]. However, determining precise adherence rates and predictors can be challenging due to small sample sizes in past studies [167].

Regarding disease-related factors, some studies found no association between adherence to the GFD and age at diagnosis [80,166]. However, others observed that at least 80% of patients diagnosed with CD before the age of 4 years adhere to the GFD, compared to only 36% of those diagnosed after 4 years of age [82].

Personality traits have also been identified as influential factors in adherence to the GFD, in addition to gender [83,159]. Studies highlighted various potentially modifiable individual factors linked to GFD adherence. These included psychological characteristics such as self-reported impulsivity [81], external locus of control [56], and poorer quality of life [168,169]. Additionally, factors like self-efficacy, risk perception, and perceived adoption of recommended behaviors play significant roles in GFD adherence [56,83,170].

However, the influence of coping strategies and personality factors on adherence to the diet remains poorly understood, which can have implications for a child’s vulnerability [53].

Adhering to a GFD presented formidable challenges encompassing multifaceted cognitive, physical, cultural, and psychological dimensions. Stringent adherence necessitates constant vigilance and careful examination of food labels, reliance on restricted product assortments (e.g., “free from” items) at retail outlets, and meticulous ingredient evaluation, leading to limited dining options in social settings, such as restaurants. Challenges intensify during transitional life phases, notably adolescence [171]. Barriers to adherence were more pronounced within peer circles compared to familial settings [161].

In the literature, it was widely acknowledged that effective coping strategies enhance emotional, physical, and social well-being as well as overall quality of life [172]. Notably, the belief in one’s capacity to influence the environment, often termed “locus of control”, was pivotal in active coping [70]. This aligns with the proposition by other authors [56] that patients with CD may develop a more internal locus of control compared to those with other chronic illnesses. Managing their disease demanded greater responsibility from CD patients than in other chronic conditions where doctors lead treatment [170].

Individuals with CD must cultivate coping strategies to maintain emotional equilibrium and psychosocial functioning, as the responsibility of managing the GFD lies with the patient or their parents. Studies focused on their adaptability to CD and the GFD [70,73]. Coping strategies may be adaptive or maladaptive, with increased task-oriented coping considered adaptive and increased emotion-oriented coping seen as maladaptive [173].

Moreover, youth with poorer adherence were more likely to report negative attitudes towards a GFD, such as feelings of isolation, embarrassment, anger, and a sense of being “different”, along with the perception that maintaining the diet was challenging [42,158]. Psychological traits and negative GFD-related attitudes may hinder effective problem-solving skills [174]. Although problem-solving specific to a GFD hasn’t been extensively studied, a qualitative study found that adolescents on a GFD who reported better dietary adherence employed multiple problem-solving strategies, such as planning ahead and bringing food to social events [155].

European studies demonstrated that culturally specific education programs, psychological support systems, and increased social awareness were effective in assisting adolescents in developing coping strategies to fully comply with a GFD during social settings with peers [67,81,154,155,156,157]. In the literature, perceived quality of life and psychological well-being emerge as crucial factors. Poor knowledge of the GFD and societal misunderstandings often exacerbated the challenges faced by individuals with CD, particularly impacting children [167]. Quality of life in children with CD is extensively studied due to its predictive value for adherence to the GFD [33,42,57,58,71,81,107]. While a GFD can alleviate symptoms, it may also impose burdens and restrictions, thereby affecting overall well-being [58,81].

Studies revealed that younger age was associated with a poorer quality of life, likely due to CD’s impact on social lifestyles [33,59]. Children with CD consistently exhibited lower emotional well-being compared to healthy counterparts, although adherence to a GFD was linked to reduced depression scores [58]. Severe symptoms significantly diminished quality of life, with psychological distress further exacerbating the issue [167].

Conversely, deteriorating quality of life increases the likelihood of abandoning the GFD, as mood alterations and quality of life disruptions may prompt discontinuation of the diet [57]. Hence, quality of life played a pivotal role in adherence to a GFD, with depressive symptoms and psychiatric conditions influencing adherence and vice versa [57].

Evaluating patients’ perspectives on illness and treatment consequences was deemed essential for a comprehensive understanding of the health–illness continuum [57,71].

### 4.5. Environmental Determinants of Vulnerability in Children with CD

Examining the literature underscored the significant influence of the family environment on adherence to GFDs, particularly within the familial context. Numerous studies underscored the profound impact of having a family member with CD on the social dynamics of those living together, with reports indicating an unconscious “withdrawal” from social life [127]. Parents often experience social isolation and stress related to caring for a child with CD, which could disrupt family dynamics and daily routines associated with adhering to GFDs. Caregivers assuming responsibility for their children’s care often reported increased depressive symptoms, family stress, and increased burden [57,127].

Typically, mothers assumed primary responsibility for purchasing and preparing gluten-free food items within the family. Their level of education and knowledge equipped them to identify GF options more effectively [158]. Statistical analysis by Rimárová [83] underscored that higher compliance rates were observed when parents possessed better education and thus a broader understanding of CD, gluten-containing products, and the significance of GFDs for their child’s growth and development. Other studies corroborated this, linking parental knowledge of GFDs with improved adherence [76,158]. Therefore, health professionals should recognize and value parental knowledge, attitudes, and beliefs in the context of caring for children with CD. Myleus [80] found that children whose parents possessed a good knowledge of CD and GFD treatment were more likely to adhere strictly to the diet.

Nutritional education plays a pivotal role in dietary adherence interventions. Nevertheless, it’s widely recognized that educational strategies alone are insufficient for driving dietary behavior changes [174]. A substantial body of evidence indicated that dietary non-compliance primarily arose from inadequate education about the GFD, misinformation, and the diet’s inherent complexity. Even the most motivated and well-educated patients may encounter challenges in adjusting to a GFD [76].

Research results consistently indicated a correlation between family socio-cultural attributes and adherence rates in the pediatric literature. Specifically, in the context of the GFD, high socioeconomic status (SES), which encompassed parental education level, parental employment status, and household income, along with parental knowledge, showed a consistent positive association with adherence [76,80,158,175].

Analysis of the literature emphasized the imperative of studying the socio-economic ramifications of maintaining lifelong and sustained adherence to this diet. Among the main challenges encountered by children diagnosed with CD and their families with regard to adherence to the GFD is the dual challenge of accessing economically viable but nutritionally solid GF dietary options. Equally crucial is educating parents about accessible gluten-free food alternatives [42,55]. Cost was another challenge associated with the GFD. Gluten is prevalent in Western diets. The majority of gluten-containing products, such as pasta and bread, typically undergo minimal processing for production. Bread, a ubiquitous dietary staple globally, naturally contains gluten and necessitates additional processing to extract the protein while preserving palatability. Consequently, substantial price differentials are prevalent among GF alternatives to gluten-rich foods due to the heightened processing demands [150,166,176,177,178,179,180].

Evidence suggests children and parents facing challenges with adherence to the GFD are unlikely to adhere strictly or may struggle with adherence in the future, necessitating tailored interventions [28]. Notably, effective adherence in young children was correlated with familial comprehension of the disease [18]. Family cohesion and support also played pivotal roles in facilitating adherence among children and adolescents adhering to restrictive diets [164]. While quantitative analyses haven’t explicitly explored the influence of family dynamics on adherence to the GFD, the significance of familial support in dietary adherence has been underscored [155]. Proposed interventions advocate for family-centered approaches in managing CD that foster GFD adherence while optimizing quality of life (QoL) for all stakeholders involved [142].

The research underscores the indispensable role of healthcare professionals, notably nurses, in elucidating and reinforcing the importance of lifelong adherence to a strict GF diet for children and their families [4]. Nurses can offer valuable guidance and support to children, families, and community healthcare providers regarding maintaining a healthy GF diet [4,44]. In cases where psychological distress is present, the collaborative involvement of both a dietitian and mental health professional is likely necessary to enhance adherence and overall health outcomes [167].

Effective support (regular follow-up) from parents is crucial for successfully managing childhood chronic illnesses and in facilitating the transition to adult medical care, including GFD management [57].

Several studies argued frequent follow-up and monitoring, along with educational resources, psychological support, and support groups can aid families in maintaining a GFD and provide creative ways to deal with the challenges inherent in a gluten-free lifestyle [49,62]. These groups share information about which foods to buy, where to buy them, as well as how to deal with the many challenges they are facing. Many families and children find great comfort in just knowing other people who are dealing with the same issues [49]. Parental support, through regular follow-up, plays a key role in effectively managing childhood chronic illnesses and facilitating the transition to adult medical care, including the management of the GFD [57].

Numerous studies advocated for frequent follow-up, monitoring, and access to educational resources, psychological support, and support groups to assist families in adhering to a GFD and coping with the challenges inherent in a GF lifestyle [49,62]. These support groups served as platforms for sharing information on suitable food options, purchasing locations, and strategies for addressing common obstacles. Many families and children found comfort in connecting with others facing similar challenges [49].

Nurses can facilitate opportunities for young individuals to engage in peer discussions regarding their experiences with managing comments from peers or adults in the community. Such interactions can occur in person, through social media platforms, online discussion groups, or participation in camps [55]. It is crucial to prioritize young people’s mental and emotional wellbeing, as peer dialogue promotes resilience to face the challenges of conformity in the midst of various social commitments [155]. Research indicated that factors such as the availability of GF meals in educational institutions and restaurants, financial assistance for families with a child diagnosed with CD, and enhanced disease and dietary information positively correlate with dietary adherence [28]. Thus, healthcare professionals must equip families with tools to facilitate access to gluten-free products, alleviate the financial burden associated with GFD, and address the social restrictions linked with dietary adherence [176].

The implementation of socio-political measures, particularly the improvement in food market regulations and labeling policies both in the European Union (EU) and globally, has significantly enhanced conditions for maintaining a gluten-free diet (GFD) [83]. The EU’s stringent legislation regarding food contaminant detection has notably contributed to this improvement. It is widely acknowledged that GF foods are generally more costly, and adhering to the GFD demands heightened creativity and commitment in meal preparation. Notably, in Italy, individuals diagnosed with CD can access clinical tests and GF food at no charge through the National Health Service upon receiving a biopsy-confirmed CD diagnosis [35]. Several countries, such as Portugal, offer state financial compensation to assist celiac patients in coping with the substantial costs associated with the GFD. However, this support is often deemed insufficient, particularly for economically disadvantaged families, leading to increased resistance to adhering to the diet among lower socioeconomic status (SES) populations [55,83,158,166].

Over the past two decades, there has been a notable increase in the availability of GF products, potentially positively impacting compliance with the GFD [83]. Nonetheless, limited access to GF foods in rural areas poses a significant challenge to adherence. Geographic factors can restrict families’ access to GF options in local stores or eateries, with convenience stores and budget supermarkets, typically situated in lower SES neighborhoods, offering limited GF selections [180]. Studies have indicated that transgressions in adherence are prevalent in urban areas, with a majority occurring among individuals of middle to low socioeconomic status [166]. Despite advancements in accessibility, external obstacles and barriers persist in maintaining the GFD, potentially impacting the quality of life of affected individuals [181]. Common challenges include dining out at public restaurants, attending social events, celebrating birthdays, and traveling [137,181]. To alleviate the psychological burden on children, feasible strategies include promoting awareness campaigns to improve the availability of GF food and ensuring safe meal options in school settings [79].

It is clear that the family is a crucial element in the response to the child’s health problem as well as school resources [182], and it is fundamental to look at the systems that the child is a part of. In these delicate conditions, the family, in the first place, and the social environment, in the second, become of crucial importance for the acceptance of the disease: parents should encourage their children not to hide their condition, thus contributing to increasing their self-esteem [155,156,157,158,159,160,161].

Nurses play a crucial role in fostering collaborative partnerships with families, characterized by mutual respect, trust, and focused attention during interactions. These healthcare professionals should actively support children and adolescents in assuming a proactive role in managing their illness, while also assisting parents and youth in accepting their health condition as an integral part of their lives rather than an impediment to participation in activities. Empowering the child is paramount, and it is essential for children of all ages to gain knowledge about their disease and dietary requirements alongside their family or caregivers [49,183].

Efforts should be made to ensure the social and school integration of children with chronic illnesses, including providing adequate support to integrate them fully into school and friend groups. Ensuring proper physical and psychological development is essential for their overall well-being [184].

The data indicated patients who perceive limitations in their social expression, particularly those experiencing poor integration within the school environment, were more likely to exhibit noncompliance with a GFD. Approximately half of the individuals with poor school integration were found to be non-adherent to the diet. Multivariate analysis revealed that the primary predictor of adherence to the GFD is the quality of the patient’s integration within the school environment, irrespective of their educational achievements. The ability to manage the GFD in relation to school dynamics is contingent upon individual personality traits and cultural background [18]. A positive social relationship was significantly associated with dietary adherence, with individuals free from feelings of self-constraint demonstrating better compliance compared to those experiencing occasional or persistent self-constraint [18].

Peers’ reactions and attitudes, social support networks, and social inconveniences all play crucial roles as mitigating factors influencing adherence [155]. School nurses are advised to deliver age-appropriate education to students, teachers, and school administrators regarding the symptoms and consequences of the disease, while also avoiding negative comments related to dietary restrictions. It is imperative to prevent mishaps by organizing annual meetings between parents, school headteachers, teachers, and the school nurse to disseminate information about the GFD, thereby minimizing opportunities for contamination, dietary lapses, and inadvertent exposure to gluten [18].

Consideration of sociocultural factors is fundamental. Membership in a CD-patient society [83,185] and parental communication with other parents of children following the GFD [158] have both been associated with improved adherence. Thus, seeking support from GFD-specific sources and community groups is vital for bolstering adherence efforts [77].

During social-dining experiences and similar gatherings with peers, adolescents are compelled to focus on food ingredients and preparation rather than the social aspects of the event itself [155]. Therefore, there is a pressing need for increased social awareness and promotion of ongoing education and support groups to assist adolescents in developing effective coping strategies that enhance compliance with the diet during social interactions outside the familial setting [67].

## 5. Conclusions

These findings illustrated the profound vulnerability of this population and underscored the necessity for comprehensive strategies to mitigate these risks. The comprehensive review synthesized the current literature on the vulnerability of children with CD, emphasizing the interconnected roles of compliance to a GFD, perceived quality of life, and lifestyle changes. It is widely acknowledged that adherence to a GFD is crucial for alleviating symptoms, supporting normal growth, and preventing long-term complications. However, compliance remains a significant challenge, particularly among children and adolescents. Factors contributing to noncompliance include a lack of awareness about the diet; limited availability of GF options; social pressures, especially among teenagers; and the palatability of GF products.

The dietary restrictions necessary for managing CD often led to substantial psychological and social challenges, impacting the quality of life and social integration of affected children. Addressing these challenges requires more than medical treatment for CD; it necessitates holistic interventions that enhance the quality of life through improved health education about a GFD, both at home and within the broader community.

Our findings highlighted the critical need for interventions that address both individual determinants—such as knowledge and behavior towards the GFD—and environmental determinants, including family, community support, and healthcare system interactions. Effective interventions should be multifaceted, aiming to reduce vulnerability by enhancing dietary compliance and social adaptation of children with CD.

To advance the management of CD, future research should focus on developing robust assessment tools that can stratify the risk of vulnerability in children with CD. These tools should take into account both individual and environmental factors to tailor nursing and healthcare interventions more effectively. By improving our understanding of these determinants, we can better target interventions to reduce the burden of CD and improve the lives of affected children.

## Figures and Tables

**Table 1:** Summary of dimensions of vulnerability in children with CD.

Vulnerability	Adverse Health Outcomes
Physical health	Implications for the child’s physical development: weight and BMI Growth failure and low height, weight, and BMI [28,29,30,31,32]
	Anemia and delayed puberty [28,29,33,34]
	Increased risk of epilepsy [35]
	Low bone mineral density [29,33,36,37]
	Nutritional risk and energy deficiencies [29,33,38,39,40,41]
	Unhealthy lifestyle diet: poor diet quality [42,43,44] with risks including obesity and cardiovascular disease [45,46,47]
	Oral health consequences [29,33,48]
	Increase of symptoms of the disease, in the case of non-compliance [32,34,49]
	Increased risk of developing other autoimmune diseases: hypothyroidism and to some extent T1DM, elevated transaminase levels [34,50] Negative impact on metabolic control, growth, and nutritional status (associated with other comorbidities (e.g., T1DM) [51,52]
Mental health	Low self-esteem [4,53] The sense of self-constraint related to the GFD [18]
	Stigma [4,18,54]
	Emotional and behavioral problems [53,55]
	Lower QoL for children and their caregivers [28,54,56,57,58,59,60,61]
	Negative implications on adherence [56]
	Increased risk for developing eating disorders (EDs) [62,63,64,65] and predominant in females [63,64,66] Increased risk of anxiety and depression [53,58,62,67] Increased susceptibility to subsequent psychiatric disorders, which persist into adulthood [29,59,62,68] Increased risk of attention deficit hyperactivity disorder (ADHD) [62] Challenges of daily disease management [55]
Social life	Changes in child’s and family members’ daily life; home and school [29,69,70] Lower levels of socialization: fewer trips to restaurants; limited leisure activities; limited travel [69] Negative implications on social integration, self-esteem, and school achievements [18,57,67,70,71] Depression and anxiety [72] Effects on parenting behaviors [72] Parenting distress/higher level of parenting stress [54,55,73]
Society	Three-fold increased risk of long-term mortality [74] Increased financial burden and challenges to care management [55]

**Table 2:** Determinants that influence, positively or negatively, vulnerability in children with CD.

Determinants	Variables	Specific Determinants That, Positively or Negatively, Influence Vulnerability in Children with CD
Individuals	Biological	Shared a genetic background that increases susceptibility to other diseases [51,52]
		Other co-morbidities: T1DM/ATD [49,52,63,75]
		Child’s developmental level: adolescence—vulnerable period [18,33,70,76,77]
	Sociodemographic	Gender [32,34,53,54]
		Age of the pediatric patient [32,75,76,78,79]
		Socioeconomic status and cultural background [47,80]
	Personality traits	Adopting internal or external locus of control (LoC) [56,70,81], persistence, harm avoidance, self-efficacy [81]
	Disease-related factors	Age of CD diagnosis [36,49,82] Time living with the disease [59] Symptoms associated with gluten ingestion [49,75] Clinical manifestations of CD [50,51,52,63]
	Acquired	Knowledge and information-seeking initiative [69,78]
		Ability to cope (include the sense and the meaning given to the chronic illness) [70,73]
		Development of effective coping strategies [44,53,67,70]
		Level of knowledge and attitude toward a GFD: adherence or non-adherence [29,39,49,53,56,58,73,78]
		Perceived quality of life and psychological well-being [53,56,57,58,59,80]
		Adoption of a new lifestyle: particular eating habits—GFD [39,44,46,62,64,68]
Environmental (Family, Community, and health system)	Parental involvement	Parenting support resulting from parental consciousness [73]
		Attitudes and beliefs: Level of perceived parental knowledge about the GFD and management of the disease [49,54,76,77,78,83]
		Parental educational status [32,83]
		Parental behavior/difficulties: parental attitudes [72]; parenting stress/burden [54,73]
	Family involvement	Family history of CD: having a first-degree relative with CD [78] Adoption of DIG in social moments by family [29]
	Social integration	Peers [78] Social awareness [62]
	School integration	Support of school health [18,29,78]
	Social resources	Existence of local support groups: membership of celiac disease patient society [44]
	Availability of GF products	Availability of gluten-free products [32,57,58,70,77,83] Family income/socioeconomic status [18]
	Accessibility to medical care and resources	Psychological support [62,68,71] Educational support/dietary counseling [4,29,39,44,45,46,47,49,52]
	Society support	Socioeconomic support measures by the National Health System [18,35]

## Appendix A

**Table A1:** Search strategy used in one of the databases.

Search	Query ^1^	Records Retrieved
#1	“vulnerab*”[Title/Abstract] OR “risk”[Title/Abstract] OR “susceptib*”[Title/Abstract]	3,168,946
#2	“celiac disease”[Title/Abstract] OR “celiac disease” [MeSH Terms] OR “coeliac disease”[Title/Abstract] OR “celiac sprue”[Title/Abstract] OR “gluten-sensitive enteropathy”[Title/Abstract])	26,851
#3	Child*[Title/Abstract] OR Child [MeSH Terms] OR adolescen*[Title/Abstract] OR adolescent [MeSH Terms] infan*[Title/Abstract] OR Infant [MeSH Terms] OR teen*[Title/Abstract] OR youth[Title/Abstract] OR scholar[Title/Abstract] OR pediatric[Title/Abstract] OR paediatric [Title/Abstract])	4,542,532
	#1 AND #2 AND #3	1778
	Limited to language (English, Portuguese, Spanish, Castilian; French)	

^1^ Search strategy for MEDLINE (via PubMed).

## Appendix B

**Table A2:** Data extraction tool.

**Details of the Scoping Review**
Title of the Scoping Review: Vulnerability in children with celiac disease: a scoping review protocol
**Goals:** To analyze the literature and map the scientific evidence regarding the vulnerability of children with celiac disease in different health care settings.
Research question: “What evidence has been published regarding the vulnerability of children with CD in different health care settings?” Sub-questions: 1. What is the published evidence on the different dimensions of vulnerability in children with CD? 2. What other concepts are related to the concept of vulnerability in children with CD? 3. Is there evidence on the use and/or validation and/or development of assessment tools on vulnerability? 4. Is there published evidence on determinants that, positively or negatively, influence vulnerability in children with CD?
Eligibility Criteria
- Participants: The review will consider studies that include school-aged (between the ages of 6 and 19) children and adolescents with CD. - Concept: Studies that explore vulnerability. - Context: Studies of a multidisciplinary nature, in different areas of expertise (hospital, primary health care, among others) will be included. No cultural or geographical restrictions.
Characteristics of the sources of evidence
Article Code/Database
Citation details (author/s, date, title, magazine, volume, editing, pages), Country, Language
Scientific discipline
Study objectives
Context
Participants (population and sample size)
Study design/Methodology/Level of evidence
Results extracted from the source of evidence
Study results (Vulnerability aspects studied/Concepts related to vulnerability/Determinants/conditions/circumstances situations that influence vulnerability)
Limitations
Future Research Recommendations/Perspectives
Bibliography cited
Comments

## Appendix C

**Table A3:** Characteristics of the articles included in this scoping review.

Authors (Year)	Discipline	Country	Background	Type of Study	Population	Assessment Instruments
Amirikian et al. [43]	Medicine	USA	Department of Hospital Pediatrics	Quantitative Study (Retrospective study)	147 (38 girls and 21 boys in the 0 to 6 age group; 36 girls and 21 boys in the 7 to 12 age group; and 23 girls and 8 boys in the 13 to 18 age group)	
Blazina et al. [186]	Medicine	Slovenia	Department of Hospital Pediatrics	Quantitative Study (Retrospective study)	55 children and adolescents (strict GFD) with negative endomysium antibodies (EMA) in the last 2 years and in 19 (not-strict GFD) with positive EMA at the time of the study	
Charalampopoulos et al. [76]	Medicine	Greece	Gastroenterology Clinic of a Children’s Hospital	Quantitative Study (Cross-sectional study)	Parents of 90 children diagnosed with CD	
Choudhary et al. [37]	Medicine	India	Department of Pediatric Medicine	Quantitative Study (Observational study)	36 children (20 females) with untreated CD at diagnosis (Group A) and 36 age- and sex-matched children on a GFD for at least one year (Group B)	
Fishman et al. [75]	Medicine	USA	Outpatient celiac clinic and support groups	Mixed-methodology study (Observational and cross-sectional study)	204 children with CD and 155 parents	
Högberg et al [82]	Medicine	Sweden	Department of Hospital Pediatrics	Quantitative Study (Observational Study)	29 adults with CD diagnosed in childhood	
Mazzone et al. [53]	Medicine	Italy	Department of Hospital Pediatrics	Quantitative Study (Cross-Sectional, comparative, and descriptive-correlational Study)	(65 females/35 males; age mean ± SD: 10.38 ± 2.71) were compared to 100 normal controls (58 females/42 males; age mean ± SD: 11.47 ± 2.61).	Emotional and behavioral problems were assessed by the Child Behavior Checklist (CBCL); Children’s Depression Inventory (CDI); Multidimensional Anxiety Scale for Children (MASC)
Kavak et al. [36]	Medicine	Turkey	Department of Hospital Pediatrics	Quantitative Study (Case-control study)	34 children with untreated CD at diagnosis and in 28 patients on a gluten-free diet for 1 year. The results were compared with those of 64 gender- and age-matched healthy control subjects	
Koziol-Kozzakowska et al. [41]	Medicine	Krakow	Department of Pediatrics, Gastroenterology, and Nutrition, University Children’s Hospital	Quantitative Study (Longitudinal study)	40 newly diagnosed CD children aged 2–18 years old	
Samasca et al. [79]	Medicine	Romania	Department of Immunology, Iuliu Hatieganu University of Medicine and Pharmacy	Qualitative Study (Narrative review)	Adolescents and young adults with CD	
Setavand et al. [31]	Nutrition and Food Sciences	Iran	Specialized Nutrition and Diet Therapy Clinic	Quantitative Study (Cross-sectional study)	Children and adolescents aged 2–18 years old (*n* = 361)	
Wagner et al. [81]	Medicine	Austria	Austrian and German Coeliac Disease Societies	Quantitative Study (Cross-sectional study)	281 children and adolescents with biopsy-proven CD (aged 10–20 years old) and 95 healthy controls	KIDCOPE—Brief Coping Checklist for Use with Pediatric Populations; Junior-Temperament and Character Inventory (J-TCI)
Bellini et al. [56]	Public Health	Italy	School	Quantitative Study (Cross-Sectional, comparative, and descriptive-correlational Study)	509 children and adolescents (aged 6–16 years old): 156 biopsy-proven with CD; 353 individuals without chronic disease	Nowicki-Strickland Locus of Control Scale (CNS-IE); QoL—Kindl Test modified
Barnes [78]	Medicine	United Kingdom	University Hospital	Qualitative Study (narrative review)	Children and young people with CD	
Cederborg et al. [69]	Psychology	Sweden	Swedish Society for Coeliacs	Qualitative Study (Phenomenological approach)	20 parents of 14 children diagnosed with CD	
Coburn et al. [60]	Medicine	USA	University School of Medicine	Qualitative Study (Systematic Review)	26 publications with study sample children under 18 years old, diagnosed with CD.	
Dogan et al. [72]	Medicine	Turkey	Department of Pediatrics, Gastroenterology, and Nutrition	Quantitative Study (Cross-sectional and comparative study)	36 children with CD (aged 4–18 years) and their mothers, and 36 healthy controls.	The Parent Attitude Research Instrument; State–Trait Anxiety Inventory; Beck Depression Inventory
De Carvalho et al. [48]	Dentistry	Brazil	Pediatric Dentistry Clinic of the School of Dentistry	Quantitative Study (Cross-sectional and comparative study)	52 children with CD and 52 controls (aged 2 to 15 years)	
Sayadi et al. [32]	Medicine	Iran	Celiac Clinic	Quantitative Study (cross-sectional study)	187 children aged between 2.5 to 14 years old with a confirmed diagnosis of CD	
Scaramuzza et al. [51]	Medicine	Italy	Department of Pediatrics	Qualitative study (narrative review)	Children with type 1 diabetes and CD	
Al-Majali, et al. [52]	Medicine	Jordan	Department of Pediatrics	Qualitative study (narrative review)	Children with type 1 diabetes and CD	
Canova et al. [63]	Public Health	Italy	Laboratory of Public Health and Population Studies—Computer data from the National Health System	Quantitative Study (retrospective, cohort, case-control study)	1215 children and young adults with CD compared with references matched for sex and year of birth.	
Conviser et al. [66]	Medicine	EUA	Department of Psychiatry and Behavioral Sciences	Qualitative Study (Systematic Review)	Empirical articles published in Medline and PsycINFO (children with CD)	
Anania et al. [46]	Medicine	Italy	Department of Pediatrics	Qualitative study (narrative review)	Epidemiological studies with children with CD	
Babio et al. [64]	Medicine	Spain	Gastroenterology Unit of University Hospital	Quantitative Study (Cross-sectional and comparative study)	98 cases of DC and 98 controls matched for gender, age, and body mass index between 10–23 years old	Children Eating Attitudes Test (ChEAT): used for children between 10 and 13 years old Eating Attitude Test (EAT)-26: over 13 years old
Clappison et al. [62]	Medicine	United Kingdom	University Hospital	Quantitative Study (Systematic Review and Meta-Analysis)	37 original articles (relationship of CD and psychiatric disorders) published in PubMed	
Lebwohl et al. [68]	Medicine	Sweden	Data from the ESPRESSO cohort.	Quantitative Study (retrospective study, a population-based cohort study)	19,186 children with a diagnosis of biopsy-verified celiac disease (from 1973 through 2016) were identified from Sweden’s 28 pathology departments	
Canova et al. [35]	Medicine	Italy	Data from the Italian National Coding System	Quantitative study (retrospective study, a population-based cohort study)	1215 individuals affected by CD and 6075 reference individuals matched by sex and age (born during 1989–2011)	
Epifanio et al. [73]	Psychology	Italy	Hospital and Pharmacy	Quantitative study (Cross-sectional and comparative study)	A group of 74 parents (28 fathers and 46 mothers, M = 37.7 years) of children (2–12 years old) with a diagnosis of CD and a group of 74 parents (22 fathers and 52 mothers, M = 35.8 years) of health children (2–12 years old)	Impact Childhood Illness Scale (ICIS); Parenting Stress Index-SF (PSI-SF).
Ballestero Fernández et al. [106]	Pharmaceutical and Health Sciences	Spain	Celiac and Gluten Sensitive Association de Madrid	Quantitative study (Cross-sectional and comparative study)	A group of 70 children and adolescents with 67 non-celiac volunteers, aged between 4 and 18, matched by sex and age.	
Di Nardo et al. [44]	Medicine	Italy	School of Medicine	Qualitative study (Systematic Review)	35 articles published in MEDLINE and EMBASE databases (children with CD)	
Errichiello et al. [18]	Medicine	Greece	Department of Pediatrics, Gastroenterology, and Nutrition	Quantitative study (cross-sectional study)	204 adolescents and young adults. Patients were divided into 2 groups: those diagnosed as children (younger than 13 years old) and those diagnosed as teenagers (older than 13 years old)	
Germone et al. [61]	Medicine	USA	Specialized care center for medical follow-up	Quantitative study (cross-sectional, comparative, and cohort study)	246 children with a confirmed diagnosis of CD and their caregivers (2–18 years old)	Pediatric Quality of Life (PedsQL) Family Impact Module (FIM)
Myléus et al. [80]	Medicine	Sweden	Databases PubMed (MEDLINE and PubMed Central), Cochrane Library, EBSCO (PsycINFO and CINAHL), and Scopus (EMBASE and MEDLINE)	Qualitative Study (Systematic Review)	49 studies of children with CD (comprising 7850 children)	
Jadresin et al. [28]	Medicine	Croatia	Department of Pediatrics, Gastroenterology, and Nutrition	Quantitative study (cross-sectional study)	71 children with a diagnosis of biopsy-verified celiac disease and their parents	
Solaymani-Dodaran et al. [74]	Health Public	United Kingdom	Division of Epidemiology and Public Health	Quantitative study (population-based cohort study)	285 children and 340 adults diagnosed with CD were followed until death, loss to follow-up, or 31 December 2004	
Rimárová et al. [83]	Medicine	Slovak Republic	Department of Public Health	Quantitative study (cross-sectional study)	325 parents or caregivers of children at age 9–15 years, with a diagnosis of CD confirmed by ESPGHAN	
Alzaben et al. [42]	Medicine	Canada	Celiac clinics at Children’s Hospital	Quantitative study (cross-sectional study)	Children and adolescents (4–18 years of age) with CD (*n* = 32) and healthy controls (*n* = 32)	
Tokatly Latzer et al. [65]	Medicine	Israel	Department of Pediatrics, Gastroenterology, and Nutrition—recruited via the National Celiac Disease Organization network	Quantitative study (population-based cohort study)	136 individuals with CD (aged 12–18 years)	Eating Attitudes Test-26 (EAT 26)
Vajro, et al. [50]	Medicine	Italy	MEDLINE/PubMed, the Cochrane Library, Web of Science, and MD Consult databases	Quantitative study (systematic review and meta-analysis)	Nine studies: 2046 children (ages 0–18 years) diagnosed by distal duodenal biopsy and/or serological tests)	
Ohlund et al. [38]	Medicine	Sweden	Department of Pediatrics	Quantitative study (cohort study)	30 children (4–17 years of age) with confirmed CD and on a GFD	
Russo et al. [142]	Nutrition	USA	Celiac Disease Center of Columbia University Irving Medical Center	Qualitative study (phenomenology study)	16 families with at least one child currently following a GFD, with a biopsy-confirmed CD diagnosis ≥1 year prior	Celiac Disease Adherence Test (CDAT); Biagi Adherence questionnaire; Celiac Disease Pediatric Quality of Life (CDPQoL); Ferretti Caregiver Questionnaire.
Dehbozorgi et al. [34]	Medicine	Iran	Hospitals affiliated with Shiraz University of Medical Sciences	Quantitative study (cross-sectional study)	130 children (under 18 years old) with biopsy-confirmed CD	
Lionetti et al. [47]	Medicine	Italy	Center for Celiac Disease of the Polytechnic University	Quantitative study (case-control prospective study)	120 children with CD and 100 healthy children (age range = 4–16 years)	KIDMED Index (Mediterranean Diet Quality Index in Children and Adolescents)
Arnone and Fitzsimons [67]	Nursing	USA	EBSCO, SAGEpub, MEDLINE, and CINAHL databases	Qualitative study (narrative review)	Empirical articles published with adolescents with CD	
Holbein et al. [77]	Medicine	USA	Center for Adherence and Self-Management	Qualitative study (narrative review)	GFD-adherence studies published (from 2008 until 2015)	
Moore [54]	Anthropology	USA	Department of Anthropology	Qualitative study (content analysis)	4 Facebook pages related to gluten-free parenting and gluten-free families	
Nayar and Mahapatra [33]	Medicine	India	Pediatric gastroenterology clinic	Mix study	20 pediatric CD (7–12 years old)	CDPQoL questionnaire
Simsek et al. [58]	Medicine	Turkey	Child Psychiatry and Pediatric Gastroenterology Clinics	Quantitative study (cross-sectional and comparative study)	25 children with CD and 25 healthy controls	Beck Depression Inventory; General-Purpose Health-Related Quality of Life Questionnaire for Children (Kid-KINDL); Pediatric Quality of Life Inventory (PedsQL)
Skjerning et al. [70]	Psychology	Denmark	Children’s Hospital	Qualitative study (focus group)	7 focus groups with 23 children/adolescents and 3 parents.	
De Lorenzo et al. [71]	Medicine	Brazil	Nutrition outpatient clinic of Children´s Hospital	Quantitative study (case-control study)	33 children with CD, 63 children without CD, and of their respective parents as their parent caregivers (96 adults)	Quality of life evaluation tools: AUQUEI scale (Autoquestionnaire de l’Enfant Image´) (children); WHOQOL-BREF (Short version, developed by the WHOQOL Group of the World Health Organization)
Jericho and Guandalini [29]	Medicine	USA	Department of Pediatrics	Qualitative review (narrative review)	Empirical articles published on children with CD	
Erickson et al. [55]	Nursing	USA	Diabetes clinic of a children´s hospital	Qualitative study (qualitative interviews)	30 parents of children/adolescents with type 1 diabetes and CD	
Fidan et al. [57]	Medicine	Turkey	Department of Pediatrics	Quantitative study (case-control study)	30 children and adolescents with CD and 100 healthy children (7–18 years)	Child Depression Inventory (CDI); State-Trait anxiety Inventory for Children (STAIC)
Sevinç et al. [59]	Medicine	Turkey	Department of Pediatrics	Quantitative study (case-control study)	52 children with CD (aged 8–12 years) and 40 healthy children	Schedule for Affective Disorders and Schizophrenia for School Age Children—Present and Lifetime Version—Turkish Version (K-SADS-PL-T) Pediatric Quality of Life Inventory (PedsQL)
Sue et al. [40]	Medicine	Australia	Department of Pediatrics	Qualitative study (narrative review)	Empirical articles published on databases of MEDLINE, EMBASE, and CINAHL (children with CD)	
Soliman, et al. [30]	Medicine	Italy	Department of Pediatrics	Quantitative study (case-control study)	30 pre-pubertal children, aged 7.4 ± 2.6 years, with CD, who were on a GFD since the age of 3.2 ± 1.6 years of age (>2 years on a GFD) for the duration of 1 year	
Sharrett and Cureton [49]	Nutrition	USA	Center for Celiac Research, Growth and Nutrition Clinic	Qualitative study (narrative review)	Empirical articles published on children with CD	
Penagini et al. [39]	Medicine	Italy	Department of Pediatrics	Qualitative study (narrative review)	Empirical articles published on children with CD	
Paul et al. [4]	Nursing	United Kingdom	Department of Pediatrics	Qualitative study (narrative review)	Empirical articles published on children with CD	
Mariani et al. [45]	Medicine	Italy	Institute of Pediatric Clinical University	Quantitative study (case-control study)	47 adolescents with CD and 47 healthy aged-matched control subjects	

**Table A4:** Summary of studies observing vulnerability in children with CD.

Authors (Year)	Title of Study	Page	Implications on Physical or Psychosocial Health	Findings
Amirikian et al. [43]	Effects of the Gluten-free Diet on Body Mass Indexes in Pediatric Celiac Patients	363	Implications on lifestyles: unhealthy food habits	Teenagers may be especially vulnerable to choosing quick and easy processed gluten-free options over more healthy, natural alternatives leading to a rise in their BMIs (…) increase in the production of processed gluten-free foods on the market.
Blazina et al. [186]	Bone mineral density and importance of strict gluten free diet in children and adolescents with celiac disease	602	Low bone-mineral density (BMD)	Children and adolescents on a not-strict GFD are at increased risk for low bone-mineral density (BMD). Also, patients on a strict GFD are at risk for low BMD because of low calcium intake or vitamin D deficiency.
Choudhary et al. [37]	Bone Mineral Density in Celiac Disease	347	Low BMD	Children with CD are at risk for reduced BMD. A strict GFD significantly improves bone mineralization. Early diagnosis and treatment of CD during childhood may protect CD patients from osteoporosis.
Kavak et al. [36]	Bone mineral density in children with untreated and treated celiac disease	436	Low BMD	Children with CD are at risk for decreased BMD (…). Low BMD responds to successful dietary treatment in just 1 year (…).
Koziol-Kozzakowska et al. [41]	Changes in Diet and Anthropometric Parameters in Children and Adolescents with Celiac Disease—One Year of Follow-Up	12	Nutritional and energy deficiencies	The low intake of key nutrients for child development, such as calcium, iron, and iodine, observed before the diagnosis, did not improve after the introduction of a GFD. There was also no significant change in the implementation of the norm for energy in children following the GFD. Taking into account the increased energy needs due to inflammation and the healing process, some CD children on a GFD may have a negative energy balance.
Mazzone et al. [53]	Compliant gluten-free children with celiac disease: an evaluation of psychological distress	5	Emotional and behavioral problems	Subjects with CD self-reported an increased rate of anxiety and depression symptoms and showed higher scores in “harm avoidance” and “somatic complaints”, in the CBCL parent-report questionnaire, as compared to healthy control subjects. Furthermore, gender differences could be observed in the group of CD patients, with males displaying significantly higher CBCL externalizing scores, in social, thought, and attention problems, as compared to females, who in turn showed more prominent internalizing symptoms such as depression.
Samasca et al. [79]	Challenges in gluten-free diet in coeliac disease: Prague consensus	4	Psychosocial burden	CD diagnosis and management can negatively affect the adolescent, impacting his/her physical and mental development, responsibilities, balanced social and behavioral life, school attendance, health management, identity confusion, risk-taking, choice selection, authority acceptance, and nutritional restrictions are imposed on potentially inadequate emotional, corporal and cognitive capabilities of the adolescent. Limited life experience, a sense of adolescent invincibility, and reluctance to abandon their pediatric "greenhouse" resulting in a sense of fear or anxiety, are some of the adolescent-period difficulties, facing transition.
Setavand et al. [31]	Evaluation of malnutrition status and clinical indications in children with celiac disease: a cross-sectional study	3–6	Growth failure and low height, weight, and BMI	Based on the CDC’s criteria, the results clearly indicated that growth failure and low height, weight, and BMI were prevalent among the children with CD. Moreover, in addition to gastrointestinal symptoms, a considerable number of patients had skeletal pain and anemia.
Wagner et al. [81]	Coeliac disease in adolescence: Coping strategies and personality factors affecting compliance with gluten-free diet	13–16	Unfavorable coping strategies in those not adhering to a GFD	In adolescents with CD, adherence to a GFD is related to unfavorable coping strategies and personality traits. This should be considered in the management of patients with CD, particularly in those not adherent to a GFD.
Bellini et al. [56]	Compliance with the Gluten-Free Diet: The Role of Locus of Control in Celiac Disease	464–465	More internal or external LoC Effects on QoL and adherence	Subjects with CD with good dietary compliance had a more internal LoC compared with those who were not compliant (*p* = 0.01). Patients who reported a satisfactory QoL had a more internal LoC compared with those who reported negative effects on QoL due to CD (*p* = 0.01).
Barnes [78]	An introduction to the management of paediatric patients with coeliac disease	45	Psychosocial experiences	Children and young people with CD present with unique challenges for the health-care team.
Cederborg et al. [69]	Living with children who have coeliac disease: a parental perspective	484–489	Changes in child’s and family’s daily life Lower levels of socialization Anxiety, fear and distrust	Implications for the child’s and family’s daily life (home and school); lower levels of socialization (fewer trips to restaurants; limited leisure activities; limited travel); need to control the type of food they offer their children; parents are unable to prevent their children from feeling different from others; concerns about their children’s future.
Coburn et al. [60]	Psychological Comorbidities in Childhood Celiac Disease: A Systematic Review	E31, 32	Psychological vulnerability and psychosocial burden Lower QoL	Studies tend to indicate elevated rates of psychological comorbidities and lower QoL in children with CD.
Dogan et al. [72]	Evaluation of the Depression, Anxiety Levels and Attitudes of Mothers of Children with Celiac Disease	372–373	Depression and anxiety maternal Effects on parenting behaviors	The mothers of children with CD had significantly higher scores in depression and state-trait anxiety than the mothers of healthy children. Mothers of children with CD had significantly higher scores in the attitude of overparenting, authoritarian attitude, and attitude of hostility and rejection than the mothers of healthy children.
De Carvalho et al. [48]	Oral aspects in celiac disease children: clinical and dental enamel chemical evaluation	1	Oral health implications	Children with CD had more recurrent aphthous stomatitis, dental enamel defects, reduced salivary flow, and chemical changes in the enamel.
Sayadi et al. [32]	Predictors of Compliance to Gluten-Free Diet in Children with Celiac Disease	2–6	In the case of non-compliance, an increase in the symptoms of the disease. Implications for the child’s physical development/health	About 40% of children adhered to a GFD poorly. This group significantly complained of more symptoms than the group with high adherence. The mean current weight and weight at the time of diagnosis as well as the mean current BMI and BMI at the time of diagnosis in the non-adherent group were significantly lower than the adherent group
Scaramuzza et al. (2013) [51]	Type 1 diabetes and celiac disease: The effects of gluten free diet on metabolic control	130	Difficulties in managing diet in the presence of other co-morbidities	This dietary restriction may be particularly difficult for the child with diabetes (…) A GFD may be rich in high glycemic index foods that can increase the risk of obesity, insulin resistance, and cardiovascular disease, worsening the metabolic control of the child with diabetes.
Al-Majali, et al. [52]	Dietary Management of Type 1 Diabetes Mellitus with Celiac Disease		Other comorbidities with impact on dietary controls	CD is diagnosed more commonly with T1DM, and the most problematic aspect for a child with T1DM and CD is that most GF foods have a high glycemic index, while low glycemic index foods are recommended for T1DM
Canova et al. [63]	Celiac Disease and Risk of Autoimmune Disorders: A Population-Based Matched Birth Cohort Study		Other comorbidities with impact on the health of the child	Children and youth with CD are at increased risk of developing autoimmune hypothyroidism and to some extent T1DM.
Conviser et al. [66]	Are children with chronic illnesses requiring dietary therapy at risk for disordered eating or eating disorders? A systematic review	1, 22	Consequences on child’s mental and physical health	Children with CD have a greater risk for developing eating disorders (EDs) than the general population and the risk is predominant in females (….) EDs and unhealthy weight management practices put children at risk for poor medical outcomes.
Anania et al. [46]	Cardiometabolic risk factors in children with celiac disease on a gluten-free diet	143	Adverse effects on body weight and cardiometabolic risk factors.	Recent epidemiological studies suggest that a GFD may have adverse effects on body weight, serum lipid levels, and insulin resistance in youths with CD.
Babio et al. [64]	Risk of Eating Disorders in Patients with Celiac Disease	53–57	Other comorbidities: Risk of eating disorders	Although being a patient with CD was associated with a significantly higher EAT (Eating Attitude Test) score in individuals above 13 years old, no clear differences were observed between individuals with CD and controls in terms of risk of an ED when other screening tests were used. More studies with larger samples and prospective designs are warranted to confirm these findings.
Clappison et al. [62]	Psychiatric Manifestations of Coeliac Disease, a Systematic Review and Meta-Analysis		Other comorbidities: Adverse effects on mental health and social life	CD is associated with an increased risk of depression, anxiety, and eating disorders as well as autism spectrum disorders (ASD) and attention deficit hyperactivity disorder (ADHD) amongst the CD population compared to healthy controls.
Lebwohl et al. [68]	Psychiatric Disorders in Patients with a Diagnosis of Celiac Disease During Childhood From 1973 to 2016		Other comorbidities: risk of psychiatric disorders	Childhood CD was associated with a 19% increase in risk of any psychiatric disorder; the increase in risk was observed in all childhood age groups. (…) Childhood CD is associated with an increased risk of subsequent psychiatric disorders, which persist into adulthood.
Canova et al. [35]	The risk of epilepsy in children with celiac disease: a population-based cohort study		Other comorbidities: risk of epilepsy	Sensitivity analyses confirmed the positive association between CD and epilepsy (…). Children and youths with CD were at increased risk of epilepsy.
Epifanio et al. [73]	Parenting stress and impact of illness in parents of children with coeliac disease	81–84	Parenting distress Higher level of parenting stress	Results evidenced a higher level of parenting stress in parents of CD children than in parents of healthy children. CD, if suitably managed, does not have a critical impact on parenting perception.
Di Nardo et al. [44]	Nutritional Deficiencies in Children with Celiac Disease Resulting from a Gluten-Free Diet: A Systematic Review	7	Implications on lifestyles: unhealthy food habits—option for processed GF food)	Children are, regardless of whether they are on a gluten-free diet or not, at risk of consuming too much fat and insufficient fiber, iron, vitamin D, and calcium. These imbalances may be exacerbated when children are on a GFD. In particular, the intake of folate, magnesium, zinc, and foods with a high glycemic index in children with CD who are on a GFD is significantly altered.
Errichiello et al. [18]	Celiac disease: predictors of compliance with a gluten-free diet in adolescents and young adults	54,	Negative implications on social integration, self-esteem, and school achievements	Children who have long accepted a GFD often rebel during adolescence, and a sizeable proportion will stop their GFD. Social integration, self-esteem, and school achievements are at risk in teenagers with CD and are likely to generate more problems than clinical complaints.
Germone et al. [61]	Family ties: the impact of celiac disease on children and caregivers	2107	Implications on HRQoL on children and caregivers	CD is associated with low HRQoL scores for both children and their caregivers. Screening children and families for HRQoL can identify patients and families in need of additional support in this higher-risk population.
Myléus et al. [80]	Rate, Risk Factors, and Outcomes of Nonadherence in Pediatric Patients With Celiac Disease: A Systematic Review	570	Implications on patient growth, current symptoms, and QoL	We found a substantial variation in the rate of adherence, ranging from 23% to 98% (…).
Jadresin et al. [28]	Compliance with gluten-free diet in children with coeliac disease	347	In children noncompliers: Implications on children’s BMI, physical development and health and quality of life	Apart from chronic fatigue in patients on a partial diet (*p* = 0.05), patient groups did not differ significantly in the frequency of symptoms. Anemia and delayed puberty were recorded only in noncompliers (*p* < 0.01 and *p* < 0.05, respectively). Noncompliers often found the specific diet posed a major life burden (*p* < 0.01) and did not visit a gastroenterologist on a regular basis (*p* < 0.01).
Solaymani-Dodaran et al. [74]	Long-term mortality in people with celiac disease diagnosed in childhood compared with adulthood: a population-based cohort study	864	Increased mortality rate.	Children diagnosed with CD had a threefold increased risk of long-term mortality
Alzaben et al. [42]	Assessing Nutritional Quality and Adherence to the Gluten-free Diet in Children and Adolescents with Celiac Disease	62	Implications for long-term health risks including obesity and cardiovascular disease in CD patients	Children with CD had higher intakes of fiber, GI, and GL and lower intakes of folate than healthy children. This was particularly evident in children with CD who were adherent to the GFD, indicating that adherence to the GFD may lead to poor diet quality due to the higher energy, fat, simple sugar, and GI and GL density in GF foods
Tokatly Latzer et al. [65]	Disordered eating behaviors in adolescents with celiac disease	365	Risk of disordered eating behaviors	EDs were found in 19% of female and 7% of male responders. These individuals were characterized by being overweight (*p* = 0.02), older age (*p* = 0.04), and female sex (*p* = 0.06).
Vajro, et al. [50]	Pediatric celiac disease, cryptogenic hypertransaminasemia, and autoimmune hepatitis		Other diseases associated with common genetic basis	CD is associated with elevated transaminase levels in about one-third of newly diagnosed children. Cryptogenic persistent HTS may signal gluten-dependent nonspecific mild hepatitis (12.0% of cases) or more rarely (6.3%) severe CD-related autoimmune hepatopathy.
Ohlund et al. [38]	Dietary shortcomings in children on a gluten-free diet	294	Nutritional risk	Children on a GFD appear to follow the same trends as healthy children on a normal diet, with high intakes of saturated fat and sucrose and low intakes of dietary fiber, vitamin D, and magnesium compared to recommendations.
Russo et al. [142]	Impact of a Child’s Celiac Disease Diagnosis and Management on the Family	2962–2968	CD’s impact on family members	Mothers and fathers rated the effects of their child’s CD differently, with mothers reporting more lifestyle changes and heavier burden (…). Mothers felt the burden of managing a gluten-free diet. Fathers felt guilty for carrying a celiac disease-associated gene and both fathers and siblings regretted limited food choices at restaurants and home. (…) Siblings felt they had developed empathy for others.
Dehbozorgi et al. [34]	Clinical manifestations and associated disorders in children with celiac disease in southern Iran	256	Other comorbidities: impact on physical health	The most common extra-intestinal manifestations included bone pain, long-term fatigue, and anemia. (…). The most common comorbidities were T1DM and hypothyroidism.
Lionetti et al. [47]	Nutritional Status, Dietary Intake, and Adherence to the Mediterranean Diet of Children with Celiac Disease on a Gluten-Free Diet: A Case-Control Prospective Study	8	Implications on lifestyles: unhealthy food habits and social impact	In the CD group, the daily intake of fats was significantly higher while the consumption of fiber was lower in comparison with the control group (…). The diet of children with CD in this study was nutritionally less balanced than controls, with a higher intake of fat and a lower intake of fiber, highlighting the need for dietary counseling.
Arnone and Fitzsimons [67]	Adolescents with celiac disease: A literature review of the impact developmental tasks have on adherence with a gluten-free diet	251–252	Psychosocial impact and stigma	Dealing with CD compounds this normal developmental stage because, in an effort to maintain peer conformity, an adolescent’s disclosure of their condition and inability to eat the same foods would make him or her visibly different. During the dining experience and similar social situations with their peers, the adolescent is consciously forced to think about food ingredients and food preparation, instead of focusing on the social aspect of the dining experience itself. When risk-taking is paired with the anxiety created by living with CD, there is an escalation in adolescents’ uncertainty, which impacts their identity formation and ultimately their compliance with a GFD. It has been demonstrated through a survey of the literature that there is a psychosocial impact on adolescents with CD and the stigma experienced by adolescents. This impact affects the rate of compliance with the diet during social situations with peers.
Moore [54]	Food Intolerant Family: Gender and the Maintenance of Children’s Gluten-Free Diets	463	Burden on mothers	Maintaining a child’s diet increases mothers’ physical, emotional, relational, and mental labor. It is argued that the gendered labor of diet management intersects with diet criticism to create a backlash rooted in gender stereotypes. Mothers face criticism for intensive, risk-averse mothering practices, yet are expected to parent intensively
Nayar and Mahapatra [33]	Nutritional Intake, Gluten-Free Diet Compliance and Quality of Life of Pediatric Patients with Celiac Disease	79, 82	Impact on physical development and growth and QoL	Pediatric CD patients non-compliant with the GFD reported a lower quality of life on the CDQoL questionnaire. Those patients who had frequent GFD transgressions had a poorer QoL (…) The trend of lower adequacy of energy observed in these patients indicates that these children are likely to suffer from protein energy malnutrition (PEM). Also, lower adequacy of calcium and riboflavin shows that these patients are susceptible to calcium deficiency (osteomalacia) and riboflavin deficiency leading to angular stomatitis, glossitis, and cheilosis. Iron and vitamin B12 deficiency together may lead to anemia in these patients
Simsek et al. [58]	Effects of Gluten-Free Diet on Quality of Life and Depression in Children With Celiac Disease	306	Implications on QoL	Patients with CD had lower QoL compared with the control group, and the low levels of dietary compliance may increase their risk for depression.
Skjerning et al. [70]	Health-related quality of life in children and adolescents with celiac disease: patient-driven data from focus group interviews	1883	Different ways of coping with CD and GFD	CD had varying impacts on the children and adolescents HRQOL. Two major categories emerged with importance for HRQOL in children and adolescents with CD, having CD (constructed from the six subcategories: symptoms, the diagnosis-process, self-perception, awareness of CD, social and emotional impact of CD, and thoughts about the future) and coping with CD (constructed from the two subcategories: coping with food and coping with social situations).
De Lorenzo et al. [71]	Evaluation of the quality of life of children with celiac disease and their parents: a case-control study	77	Impact on QoL	There is an impairment of the QoL of children with CD and of their parents, with regard to social life, particularly in the leisure (children) and social (adults) dimensions.
Jericho and Guandalini [29]	Extra-Intestinal Manifestation of Celiac Disease in Children	1–11	Extra-intestinal manifestations of disease	The extra-intestinal manifestations of CD seen most often in the pediatric population include (…) short stature, delayed puberty, dental enamel hypoplasia, osteopenia/osteoporosis, iron-deficiency anemia refractory to oral iron supplementation, recurrent stomatitis, liver and biliary disease, dermatitis herpetiformis, arthralgia/arthritis, headaches, ataxia, peripheral neuropathy, epilepsy, behavioral changes, psychiatric disorders, and alopecia.
Erickson et al. [55]	Parent Experiences Raising Young People with Type 1 Diabetes and Celiac Disease	353–363	Negative health consequences, increased financial burden, and challenges to care management	Analysis revealed six themes: (a) health complications of T1D, (b) challenges of daily disease management, (c) financial concerns, (d) the young person’s emotional/mental health, (e) experiences with healthcare providers, and (f) experiences with people outside the family and at school
Fidan et al. [57]	Depression-anxiety levels and the quality of life among children and adolescents with coeliac disease	232, 237	Impact on QoL	Results indicated that children and adolescents with CD were negatively affected in terms of the psychological and social quality of life during with a chronic disease.
Sevinç et al. [59]	Psychopathology, quality of life, and related factors in children with celiac disease	1	Impact on QoL and contribution to the development of psychiatric disorders	CD is associated with some psychiatric signs/diagnoses, and it decreased QoL. When the possible factors that cause these results were investigated, it was found that these outcomes are related to being female and the decrease in some parts of QoL due to the duration of the disease. On the other hand, according to these findings, both impaired QoL and increased psychopathologies were not related to worse compliance with a GFD.
Sue at al [40]	Paediatric Patients with Coeliac Disease on a Gluten-Free Diet: Nutritional Adequacy and Macro and Micronutrient Imbalances	9	Nutritional risk with an impact on physical development; Impact on alimentary patterns	The current literature demonstrates that, whether on a GFD or not, children are at risk of consuming excess fat and insufficient fiber, iron, vitamin D, and calcium. These imbalances may be worsened on a GFD, as in the case of fat, or have implications that are even more important in a patient with CD, as in the case of vitamin D and calcium. Children with CD on a GFD may have significantly altered intake of folate, magnesium, zinc, selenium, and foods with a high glycemic index.
Soliman, et al. [30]	Linear growth of children with celiac disease after the first two years on gluten-free diet: A controlled study	20	Impact on physical development and growth	Most of our children with CD grew normally both in height and weight while following the GFD. Significant catch-up growth occurred in some of them after 2 years of being on a GFD.
Sharrett and Cureton [49]	Kids and the gluten-free diet	1	Risk of complications of untreated CD	Upon accurate diagnosis and treatment, children usually improve quickly; however, despite rapid improvement of symptoms, compliance with diet may be less than optimal, putting the child once again at risk for complications of untreated CD.
Penagini et al. [39]	Gluten-free diet in children: Health benefits and nutritional complications		Impact on physical development and growth	A GFD, if not carried out with attention, may paradoxically lead to nutritional imbalances, which should be avoided, particularly at the pediatric age, the phase of maximal growth and development
Paul et al. [4]	Diagnosis and nursing management of coeliac disease in children	24	Impact on self-esteem on adherence	Children and adolescents may experience low self-esteem due to dietary restrictions identifying them as being different from their peers, and this can negatively affect their adherence to a GFD.
Mariani et al. [45]	The gluten-free diet: a nutritional risk factor for adolescents with celiac disease?		Nutritional risk	Adherence to a strict GFD worsens the already nutritionally unbalanced diet of adolescents, increasing elevated protein and lipid consumption.

**Table A5:** Summary of studies addressing concepts related to vulnerability in children with CD.

Authors (Year)	Title of Study	Concepts
Amirikian et al. [43]	Effects of the Gluten-free Diet on Body Mass Indexes in Pediatric Celiac Patients	Lifestyles
Di Nardo et al. [44]	Nutritional Deficiencies in Children with Celiac Disease Resulting from a Gluten-Free Diet: A Systematic Review	Lifestyles
Anania et al. [46]	Cardiometabolic risk factors in children with celiac disease on a gluten-free diet	Lifestyles; compliance with a GFD
Fishman et al. [75]	Creation of Experience-based Celiac Benchmarks: The First Step in Pretransition Self-management Assessment	Self-management
Charalampopoulos et al. [76]	Determinants of adherence to gluten-free diet in Greek children with coeliac disease: a cross-sectional study	Compliance with a GFD
Mazzone et al. [53]	Compliant gluten-free children with celiac disease: an evaluation of psychological distress	Compliance with a GFD, QoL, coping
Samasca et al. [79]	Challenges in gluten-free diet in coeliac disease: Prague consensus	Compliance with a GFD
Wagner et al. [81]	Coeliac disease in adolescence: Coping strategies and personality factors affecting compliance with gluten-free diet	Coping Personality traits (LoC; self-efficacy) Compliance with a GFD
Bellini et al. [56]	Compliance with the Gluten-Free Diet: The Role of Locus of Control in Celiac Disease	Compliance with a GFD; quality of life (QoL) Personality traits: (LoC)
Barnes [78]	An introduction to the management of paediatric patients with coeliac disease	Compliance with a GFD
Cederborg et al. [69]	Living with children who have coeliac disease: a parental	Compliance with a GFD; attitudes and beliefs
Dogan et al. [72]	Evaluation of the Depression, Anxiety Levels and Attitudes of Mothers of Children with Celiac Disease	Parental attitude, parental stress
Sayadi et al. [32]	Predictors of Compliance to Gluten-Free Diet in Children with Celiac	Compliance with a GFD
Scaramuzza et al. (2013) [51]	Type 1 diabetes and celiac disease: The effects of gluten free diet on metabolic control	Compliance with a GFD
Al-Majali, et al. [52]	Dietary Management of Type 1 Diabetes Mellitus with Celiac Disease	Compliance with a GFD
Babio et al. [64]	Risk of Eating Disorders in Patients with Celiac Disease	Compliance with a GFD; body-image
Clappison et al. [62]	Psychiatric Manifestations of Coeliac Disease, a Systematic Review and Meta-Analysis	QoL
Epifanio et al. [73]	Parenting stress and impact of illness in parents of children with coeliac disease	QoL; Parental Stress; coping; personality traits: (resilience)
Ballestero Fernández et al. [106]	Nutritional Status in Spanish Children and Adolescents with Celiac Disease on a Gluten Free Diet Compared to Non-Celiac Disease Controls	Compliance with a GFD; lifestyles
Errichiello et al. [18]	Celiac disease: predictors of compliance with a gluten-free diet in adolescents and young adults	Compliance with a GFD, QoL
Germone et al. [61]	Family ties: the impact of celiac disease on children and caregivers	HRQoL (health-related quality of life)
Myléus et al. [80]	Rate, Risk Factors, and Outcomes of Nonadherence in Pediatric Patients with Celiac Disease: A Systematic Review	Compliance with a GFD; QoL; knowledge, attitudes and beliefs; personality traits (LoC)
Tokatly Latzer et al. [65]	Disordered eating behaviors in adolescents with celiac disease	Compliance with a GFD; body image
Jadresin et al. [28]	Compliance with gluten-free diet in children with coeliac disease	Compliance with a GFD; attitudes and beliefs; QoL
Solaymani-Dodaran, M. et al. (2007) [74]	Long-term mortality in people with celiac disease diagnosed in childhood compared with adulthood: a population-based cohort study.	Lifestyles; coping
Rimárová et al. [83]	Compliance with gluten-free diet in a selected group of celiac children in the Slovak Republic	Compliance with a GFD
Alzaben et al [42]	Assessing Nutritional Quality and Adherence to the Gluten-free Diet in Children and Adolescents with Celiac Disease	Lifestyles; compliance with a GFD
Ohlund et al. [38]	Dietary shortcomings in children on a gluten-free diet	Lifestyles; compliance with a GFD
Russo et al. [142]	Impact of a Child’s Celiac Disease Diagnosis and Management on the Family	QoL, stress/burden parental; compliance with a GFD.
Lionetti et al. [47]	Nutritional Status, Dietary Intake, and Adherence to the Mediterranean Diet of Children with Celiac Disease on a Gluten-Free Diet: A Case-Control Prospective Study	Lifestyles; compliance with a GFD
Arnone and Fitzsimons [67]	Adolescents with celiac disease: A literature review of the impact developmental tasks have on adherence with a gluten-free diet	Compliance with a GFD; body image; stigma; lifestyles; QoL; coping
Holbein, et al. [77]	Topical Review: Adherence Interventions for Youth on Gluten-Free Diets	Compliance with a GFD; self-management; coping; personality traits (self-efficacy)
Moore [54]	Food Intolerant Family: Gender and the Maintenance of Children’s Gluten-Free Diets	Compliance with a GFD; lifestyles; parental stress/burden
Nayar and Mahapatra [33]	Nutritional Intake, Gluten-Free Diet Compliance and Quality of Life of Pediatric Patients with Celiac Disease	Compliance with a GFD; QoL; lifestyles
Simsek et al. [58]	Effects of Gluten-Free Diet on Quality of Life and Depression in Children with Celiac Disease	Compliance with a GFD; QoL
Skjerning et al. [70]	Health-related quality of life in children and adolescents with celiac disease: patient-driven data from focus group interviews	Compliance with a GFD; HRQoL; coping
De Lorenzo et al. [71]	Evaluation of the quality of life of children with celiac disease and their parents: a case-control study	QoL; coping
Erickson et al. [55]	Parent Experiences Raising Young People with Type 1 Diabetes and Celiac Disease	Parental stress/burden; self-management; lifestyles
Coburn et al. [60]	Psychological Comorbidities in Childhood Celiac Disease: A Systematic Review	QoL
Fidan et al. [57]	Depression-anxiety levels and the quality of life among children and adolescents with coeliac disease	QoL
Sevinç et al. [59]	Psychopathology, quality of life, and related factors in children with celiac disease	QoL, Compliance with a GFD
Jericho and Guandalini [29]	Extra-Intestinal Manifestation of Celiac Disease in Children	Compliance with a GFD
Soliman et al. [30]	Linear growth of children with celiac disease after the first two years on gluten-free diet: A controlled study	Compliance with a GFD
Kavak et al. [36]	Bone mineral density in children with untreated and treated celiac disease	Compliance with a GFD
Blazina et al. [186]	Bone mineral density and importance of strict gluten free diet in children and adolescents with celiac disease	Compliance with a GFD
Choudhary et al. [37]	Bone Mineral Density in Celiac Disease	Compliance with a GFD
Koziol-Kozzakowska et al. [41]	Changes in Diet and Anthropometric Parameters in Children and Adolescents with Celiac Disease—One Year of Follow-Up	Compliance with a GFD
Sue at al [40]	Paediatric Patients with Coeliac Disease on a Gluten-Free Diet: Nutritional Adequacy and Macro and Micronutrient Imbalances	Lifestyles
Sharrett and Cureton [49]	Kids and the gluten-free diet	Compliance with a GFD; lifestyles; stigma
Penagini et al. [39]	Gluten-free diet in children: Health benefits and nutritional complications	Compliance with a GFD; lifestyles
Paul et al. [4]	Diagnosis and nursing management of coeliac disease in children	Compliance with a GFD; stigma; QoL
Mariani et al. [45]	The gluten-free diet: a nutritional risk factor for adolescents with celiac disease?	Compliance with a GFD; lifestyles

Appendix C (Table A6) reported the data presentation template for Question 4.

**Table A6:** Summary of studies observing determinants that, positively or negatively, influence vulnerability in children with CD.

Authors (Year)	Title of Study	Page	Influencing Determinants of Vulnerability in Children with CD
Charalampopoulos et al. [76]	Determinants of adherence to gluten-free diet in Greek children with coeliac disease: A cross-sectional study	618	The age of the pediatric patient and estimation of the level of perceived parental knowledge are two factors that a clinician can utilize as a "prognostic tool" in order to identify children who run a high risk of being noncompliant with a GFD and, therefore, modify their counseling strategy accordingly.
Högberg et al. [82]	Better dietary compliance in patients with coeliac disease diagnosed in early childhood	753	At least 80% of the celiac patients who had been diagnosed before the age of 4 years complied with the GFD compared to 36% of the CD patients older than 4 years at diagnosis (*p* < 0.05)
Mazzone et al. [53]	Compliant gluten-free children with celiac disease: an evaluation of psychological distress	5	Gender differences could be observed in the group of CD patients, with males displaying significantly higher CBCL externalizing scores in social, thought, and attention problems as compared to females, who in turn showed more prominent internalizing symptoms such as depression.
Kavak et al. [36]	Bone mineral density in children with untreated and treated celiac disease	436	Early diagnosis and treatment of CD, particularly during childhood, will protect patients from osteopenia.
Samasca et al. [79]	Challenges in gluten-free diet in coeliac disease: Prague consensus	4	The adolescence period, young adults, and the transition itself: The transition period brings some more reasons for poor compliance.
Bellini et al. [56]	Compliance with the Gluten-Free Diet: The Role of Locus of Control in Celiac Disease	463	Compliant CD patients who have an internal LoC tend to maintain this internality, leading to optimal self-control and good QoL—the usefulness of the LoC concept for identifying those patients who might be at risk for dietary transgression.
Barnes [78]	An introduction to the management of paediatric patients with coeliac disease	44–45	With appropriate, targeted management and support, patients can meet their health potential (if applicable, school nursing)
Cederborg et al. [69]	Living with children who have coeliac disease: A parental perspective	488–489	Information-seeking process (by parents) to find good information (the Internet is one source of information; another is an association for people with CD) Teenagers diagnosed with CD may find it more difficult to comply with a GFD, especially if they are asymptomatic when eating food containing gluten. It may be easier to adapt to a GFD for younger children because they learn early in life what food they tolerate, and they have limited experience of how a diet that contains gluten can taste.
Dogan et al. [72]	Evaluation of the Depression, Anxiety Levels and Attitudes of Mothers of Children with Celiac Disease	373	Mothers have a major influence on the well-being and adjustment of their children and play an important role in the adaptation of their children to living with an illness
Sayadi et al. [32]	Predictors of Compliance to Gluten-Free Diet in Children with Celiac Disease	4	Inaccessibility, high costs, and lack of food labeling were the primary reasons for nonadherence to a GDF. Furthermore, no significant relationship was found between following a GFD and age, age at the time of diagnosis, gender, and parental educational status.
Scaramuzza et al. (2013) [51]	Type 1 diabetes and celiac disease: The effects of gluten free diet on metabolic control	131	The increased prevalence of CD in patients with T1DM is due to an overlap in the genetic susceptibility to both diseases conferred by the HLADR3/DQ2. This haplotype is present in over 90% of patients with CD and 55% of those with T1DM, compared with only 20–25% of the general population of European ancestry. HLA-DQ8 also confers a risk of T1DM.
Al-Majali, et al. [52]	Dietary Management of Type 1 Diabetes Mellitus with Celiac Disease	1	The most problematic aspect for a child with T1DM and CD is that most GF foods have a high glycemic index, while low glycemic index foods are recommended for T1DM. As a result, dietary controls become more difficult.
Canova et al. [63]	Celiac Disease and Risk of Autoimmune Disorders: A Population-Based Matched Birth Cohort Study	6	The most plausible mechanism explaining the association between CD and T1DM/ATD is a shared genetic background.
Anania et al. [46]	Cardiometabolic risk factors in children with celiac disease on a gluten-free diet	147	Screening for cardiometabolic risk factors in celiac children is to be recommended not only at diagnosis but also during follow-up since an early intervention may prevent cardiovascular morbidity.
Babio et al. [64]	Risk of Eating Disorders in Patients with Celiac Disease	53–57	Food restrictions may focus the attention of patients with CD on food and their body image.
Clappison et al. [62]	Psychiatric Manifestations of Coeliac Disease, a Systematic Review and Meta-Analysis	12	Psychological support beyond simply advising a GFD may promote acceptance and subsequent adherence to the GFD as well as reducing the risk of anxiety and depression.
Lebwohl et al. [68]	Psychiatric Disorders in Patients with a Diagnosis of Celiac Disease during Childhood From 1973 to 2016	16–17	The psychosocial stress associated with adapting to the gluten-free diet may contribute to the increased incidence of psychiatric disorders in both the short and long term. However, this risk is unlikely to be due to the GFD alone since we also observed an increased risk of psychiatric disorders preceding the diagnosis of CD, possibly related to the systemic inflammatory response described above (…). These findings emphases the importance of not just somatic surveillance but also mental health surveillance for timely support and intervention.
Canova et al. [35]	The risk of epilepsy in children with celiac disease: A population-based cohort study	1090	In Italy, all patients with CD can obtain clinical tests and gluten-free food without charge, provided by the National Health Service, in the presence of a biopsy-verified CD diagnosis.
Epifanio et al. [73]	Parenting stress and impact of illness in parents of children with coeliac disease	84	Parenting difficulties: “regards the risk he may injure himself and it could regard the fear of the food contamination” (…) “parents are preoccupied that the illness has an impact on the child’s internal affective world and his sensitivity. The most frequent concern regards the explanation of the illness to the child himself (…) and it could be associated with parenting difficulties to find an explanation, emotionally satisfactory, of the causes of the illness and to accept profoundly its incurability” Child’s age: With the growth, children become more cognitively able to understand the meaning of being ill and the meaning of the food restrictions. However, they can become more conscious of the difference compared with peers, especially with the admission to school and in adolescence. Indeed, the adolescent prepares himself to cope with the hard body and identity transformation; a process in which he has to include the sense and the meaning to give to his chronic illness Adherence to a therapeutic regimen “has a strong influence on the growth and the health condition of the CD child”. Parenting support: “When patient is a child, the parenting support plays an important role in treatments adherence, and an optimal disease management requires the parenting consciousness of the own new role and of the change from the condition of parent to that of caregiver, that is of specific care provider”.
Ballestero Fernández et al. [106]	Nutritional Status in Spanish Children and Adolescents with Celiac Disease on a Gluten Free Diet Compared to Non-Celiac Disease Controls	21	Children and adolescents with CD following a GFD for over a year appear to follow the same trends as healthy children on a normal diet, considering the nutrient quality of the diet, anthropometric measures, biochemical biomarkers, bone mineral density, and physical activity.
Di Nardo et al. [44]	Nutritional Deficiencies in Children with Celiac Disease Resulting from a Gluten-Free Diet: A Systematic Review	8	Concrete solutions/suggestions for the daily lives of these individuals that contribute to adherence to a GFD emerged from the latest research: 1. Provide patients with the name and telephone number of any local support groups. Face-to-face help enhances compliance and feelings of empowerment and reduces feelings of isolation; 2. Provide educational materials to address the patients’ most urgent needs. The materials may need to be divided into survival skills (which of the foods are gluten-free, what foods to avoid, and where to source the foods locally), day-to-day coping (reading labels, recipes, etc.), and longer-term coping strategies (eating out and travel); 3. Set aside some time during follow-up visits to inquire about the clients’ adjustment to the gluten-free diet and lifestyle; 4. Encourage members of the patient’s family to attend follow-up visits as this provides an opportunity to discuss lifestyle adjustments; 5. Encourage any patients who seem to be having difficulties with the diet and/or compliance to make the most of support groups, social workers, or family counseling;
Errichiello et al. [18]	Celiac disease: predictors of compliance with a gluten-free diet in adolescents and young adults	3–5	All of the variables that concern family, school, and social interaction are related. But, (…) Only school integration significantly contributed to the likelihood of good or poor compliance (Wald statistics ¼ 10.83, *p* < 0.001, odds ratio 0.44). For each degree of improved school integration, we have about 56% less transgression from the GFD (…). (…) Children who do not receive from their home and school environments enough support should be paid more attention by us, the care team, and the school health system. We have to identify a red flag, which can be used to support the ‘‘weak’’ ones.
Germone et al. [61]	Family ties: the impact of celiac disease on children and caregivers	2116	Routine screening and timely access to psychosocial support are suggested to mitigate the negative impact of the condition. Care for children and families may include a multidisciplinary approach involving support for diet management from a dietitian and health behavior services from a qualified behavioral health provider.
Myléus et al. [80]	Rate, Risk Factors, and Outcomes of Nonadherence in Pediatric Patients with Celiac Disease: A Systematic Review	569–570	Among the various risk factors (sociodemographic, disease-related factors, treatment factors, knowledge/attitudes and beliefs, sociocultural and environmental factors, quality of life, and psychological well-being), adolescence is a vulnerable period, and parental knowledge about CD was associated with children’s adherence. Sociodemographic factors: (…) “adherence was lower among adolescents compared with younger children”; “the adherence appears to be comparable in girls and boys (was not found a statistically significant differences); (…) some studies suggested higher adherence among those with higher socioeconomic status; Disease-related factors: (…)”; "there is no association between adherence and age at diagnosis, family history of CD, comorbidities and symptomatic disease at presentation”; Treatment factors: “Taste of the gluten-free food was suggested to affect adherence” and “longer duration was beneficial, but then possibly declining again after 15 years.” Knowledge, attitudes, and beliefs: “Children whose parents had good knowledge about CD and the treatment with GFD were more likely to adhere strictly. Furthermore, nonadherence children believed they could be healthy without a GFD to a larger extent than adherence children did.” Sociocultural and environmental factors: “Being a member in a celiac disease patient society was associated with higher adherence. (…) No association between adherence and to community size or urban or rural habitation was found”. QoL and psychological well-being: No study investigated quality of life as a risk factor for suboptimal adherence to a GFD. One study investigated the locus of control showing that those adhering to a GFD believed to a larger extent that events are more contingent on their own behavior compared with those that are not adhering.
Jadresin et al. [28]	Compliance with gluten-free diet in children with coeliac disease	344	An active attitude is required in the follow-up of patients with CD due to the risk of developing complications later in life.
Rimárová et al. [83]	Compliance with gluten-free diet in a selected group of celiac children in the Slovak Republic	S23	Individual child factors that influence adherence to DIG: age of the child: “Young children with celiac disease adhered better to GF diet.” Child’s family factors influencing adherence to DIG: “Mother’s education was considered as a significant factor related to the GF diet compliance. Usually, the mother in the family is responsible for buying and preparation of food items”; “parents’ positive attitude towards the child’s condition is associated with higher compliance”; “higher degree of compliance is noted when parents have better education and therefore wider knowledge about celiac disease and the gluten containing products, understand the importance of gluten-free diet for their child’s overall growth and development, and are able to distinguish gluten-containing from gluten-free food.” Children’s environmental factors that influence adherence to DIG: “There has been improved availability of GF products over the last two decades, which may have positive influence on compliance with GF diet.”
Tokatly Latzer et al. [65]	Disordered eating behaviors in adolescents with celiac disease	368; 370	“We identified female gender, older age and being overweight as risk factors for DEBs in individuals with CD. (…) to the importance of closely monitoring adolescents with CD for DEBs, especially so if female, overweight or older; and if diagnosed with CD at a later age, regardless of their GFD adherence tendencies.”
Russo et al. [142]	Impact of a Child’s Celiac Disease Diagnosis and Management on the Family	2968	A child’s CD diagnosis and concomitant GFD management affects the entire family. Our results can help inform family-centered CD interventions that promote GFD maintenance while maximizing QOL for all involved.
Dehbozorgi et al. [34]	Clinical manifestations and associated disorders in children with celiac disease in southern Iran	256	Gender: “Celiac disease was detected more amongst females (63.8%)”
Lionetti et al. [47]	Nutritional Status, Dietary Intake, and Adherence to the Mediterranean Diet of Children with Celiac Disease on a Gluten-Free Diet: A Case-Control Prospective Study	9	Dietary counseling: Our study, together with a review of the literature, highlights the need for celiac patients to receive dietary counseling, a fundamental tool to teach the patient to increase the consumption of naturally gluten-free products, to reduce processed ones, to increase the intake of cereals such as oats, rice, minor and pseudo-cereals, and to adhere to the rules of the Mediterranean diet.
Arnone and Fitzsimons [67]	Adolescents with celiac disease: A literature review of the impact developmental tasks have on adherence with a gluten-free diet	252	Development of effective coping strategies through culturally specific education, social support systems, and increased social awareness are warranted for the adolescent to fully comply with the GFD in social situations.
Holbein et al. [77]	Topical Review: Adherence Interventions for Youth on Gluten-Free Diets		There are few evidence-based, published pediatric GFD adherence interventions. (...) Nonmodifiable and modifiable factors within individual, family, community, and health systems domains must be considered when developing future interventions. (…) Nonmodifiable factors (e.g., developmental level, SES, cultural background, availability of GF foods) must be considered when adapting intervention components to best fit participant characteristics. Further, modifiable influences (e.g., social support, parental knowledge about GFDs, GFD-related attitudes, and patient–provider communication) can inform the targets of adherence interventions and the processes (e.g., education, problem solving, and cognitive restructuring) incorporated into the program; it is likely multiple treatment mechanisms may be needed to sustain adherence outcomes
Moore [54]	Food Intolerant Family: Gender and the Maintenance of Children’s Gluten-Free Diets	463	Sociodemographic factors (gender): Women’s visibility in connection to gluten-free diets contributes to backlash against the diet infused with negative stereotypes of women and mothers. This results in additional burdens on mothers of gluten-free children.
Simsek et al. [58]	Effects of Gluten-Free Diet on Quality of Life and Depression in Children with Celiac Disease	306	In terms of preventing depression during the course of CD, clinicians should follow-up these patients more closely with regard to their dietary compliance.
Skjerning et al. [70]	Health-related quality of life in children and adolescents with celiac disease: patient-driven data from focus group interviews	1891	Patterns of behaviors emerging: One pattern was bringing GF food to new environments to alleviate stress about the availability of GF foods. In addition, checking the labeling of food and when unsure asking questions about the gluten content. This can be described as ‘‘primary control coping’’ as a problem-solving response to coping with CD and the GFD. Another pattern was adapting to the stressor as a means of coping. This is viewed as ‘‘accommodative or secondary control coping’’. Interestingly, it becomes apparent that this pattern of coping may have more beneficial outcomes as it involves restructuring cognitive schemas surrounding chronic illness. The use of ‘‘primary control coping’’ may result in feelings of anger, frustration, and loss of control as a response has to be addressed to each stressor by trying to change it, rather than adapting to it. A cognitive restructuring technique may lead to improved HRQOL by taking control of the perception of the GFD and adapting it to suit the requirements of this chronic disease. In contrast, experiencing that the GFD is out of control seems to be related to poorer HRQOL and lower adherence to the GFD. This emerged in a final coping strategy that involved avoiding or denying the source of stress. This pattern of coping could be called ‘‘passive or disengagement coping’.
De Lorenzo et al. [71]	Evaluation of the quality of life of children with celiac disease and their parents: a case-control study	84	Psychosocial support: Health professionals should offer both adults and children with CD-specific information, psychosocial support, and encouragement to help them plan and develop their own strategies to handle risky situations or social discomfort and amplify actions to promote opportunities for socialization and leisure.
Jericho and Guandalini [29]	Extra-Intestinal Manifestation of Celiac Disease in Children	7	Strict, lifelong adherence to a GFD remains the only available treatment for patients who are diagnosed with CD, and (…), should result in a complete return to health in the majority of patients, especially pediatric.
Erickson et al. [55]	Parent Experiences Raising Young People with Type 1 Diabetes and Celiac Disease		Educational support: “Nurses can play a critical role in helping parents understand the importance of following management protocols for both diseases in order to minimize long- and short-term complications”; “Nurses can also provide families with resources such as parent groups where issues and challenges are shared with other parents, and offer specific suggestions to overcome some of these challenges discussed”; Therefore, nurses need to be sensitive to both psychological and social challenges children/adolescents living with T1D and CD and their parents face over time; “Because both diseases are expensive, it is critical providers talk with parents regarding ways to minimize expenses such as buying diabetic supplies and gluten-free foods in bulk or from pharmacies or stores where costs are more reasonable. Educating parents about simple, readily available gluten-free foods is also important”; Emotional support: “Providing opportunities or resources for young people with both diseases to talk with other young people regarding how they respond to comments made by peers or adults in the community may also be helpful. Such interactions could be in person, through social media, online discussion groups or, attending camps for children with T1D or CD." Family and school involvement: “Since these young people need to strictly adhere to a gluten-free diet, those outside the family and at school also should be encouraged to avoid making negative comments related to this restriction. School nurses are encouraged to provide age-appropriate education to students, teachers, and school administrators related to the symptoms and effects of T1D and CD”
Fidan et al. [57]	Depression-anxiety levels and the quality of life among children and adolescents with coeliac disease		Decreased QoL can be associated with dietary restrictions and it is important to make products suitable for the diets of children and adolescents with CD are more readily and commonly available
Sevinç et al. [59]	Psychopathology, quality of life, and related factors in children with celiac disease	6	Children and adolescents diagnosed with CD should be followed up by a child psychiatrist for successful adherence to diet and, hence, optimal QoL and mental health.
Sharrett and Cureton [49]	Kids and the gluten-free diet	49	Frequent follow-up and monitoring, along with educational resources and support groups can aid families in maintaining a GFD and provide creative ways to deal with the challenges inherent in a gluten-free lifestyle.
Penagini et al. [39]	Gluten-free diet in children: Health benefits and nutritional complications	4562	Increasing awareness of the possible nutritional deficiencies associated with the GFD may help healthcare professionals and families tackle the issue by starting with early education on the GFD and clear dietary advice on how to choose the most appropriate gluten-free foods.
Paul et al. [4]	Diagnosis and nursing management of coeliac disease in children	22, 24	Children’s nurses have an important role in recognizing and diagnosing CD earlier as well as offering ongoing dietary support.
Mariani et al. [45]	The gluten-free diet: a nutritional risk factor for adolescents with celiac disease?	519–523	In the follow-up of patients with CD, considerable effort has yet to be made to improve compliance with a gluten-free diet, and especially to control the nutritional balance of the diet in compliant patients.
